# Identification of two integration sites in favor of transgene expression in *Trichoderma reesei*

**DOI:** 10.1186/s13068-018-1139-3

**Published:** 2018-05-17

**Authors:** Lina Qin, Xianzhang Jiang, Zhiyang Dong, Jianzhong Huang, Xiuzhen Chen

**Affiliations:** 1 National and Local Joint Engineering Research Center of Industrial Microbiology and Fermentation Technology, College of Life Sciences, Fujian Normal University, Qishan Campus, No.1 Keji Road, Shangjie, Minhou, Fuzhou, 350117 Fujian China; 2 State Key Laboratory of Microbial Resources, Institute of Microbiology, Chinese Academy of Sciences, Beijing, 100101 China; 3 Provincial University Key Laboratory of Cellular Stress Response and Metabolic Regulation, College of Life Sciences, Fujian Normal University, Fuzhou, 350117 Fujian China

**Keywords:** 2A peptide, Flow cytometry, *Trichoderma reesei*, Integration site, Lipase, Gene expression

## Abstract

**Background:**

The ascomycete fungus *Trichoderma reesei* was widely used as a biotechnological workhorse for production of cellulases and recombinant proteins due to its large capacity of protein secretion. Transgenesis by random integration of a gene of interest (GOI) into the genome of *T. reesei* can generate series of strains that express different levels of the indicated transgene. The insertion site of the GOI plays an important role in the ultimate production of the targeted proteins. However, so far no systematic studies have been made to identify transgene integration loci for optimal expression of the GOI in *T. reesei*. Currently, only the locus of exocellobiohydrolases I encoding gene (*cbh1)* is widely used as a promising integration site to lead to high expression level of the GOI. No additional sites associated with efficient gene expression have been characterized.

**Results:**

To search for gene integration sites that benefit for the secreted expression of GOI, the food-and-mouth disease virus 2A protein was applied for co-expression of an *Aspergillus niger lipA* gene and *Discosoma* sp. *DsRed1* gene in *T. reesei,* by random integration of the expression cassette into the genome. We demonstrated that the fluorescent intensity of RFP (red fluorescent protein) inside of the cell was well correlated with the secreted lipase yields, based on which, we successfully developed a high-throughput screening method to screen strains with relatively higher secreted expression of the GOI (in this study, lipase). The copy number and the insertion sites of the transgene were investigated among the selected highly expressed strains. Eventually, in addition to *cbh1* gene locus, two other genome insertion loci that efficiently facilitate gene expression in *T. reesei* were identified.

**Conclusions:**

We have successfully developed a high-throughput screening method to screen strains with optimal expression of the indicated secreted proteins in *T. reesei*. Moreover, we identified two optimal genome loci for transgene expression, which could provide new approach to modulate gene expression levels while retaining the indicated promoter and culture conditions.

**Electronic supplementary material:**

The online version of this article (10.1186/s13068-018-1139-3) contains supplementary material, which is available to authorized users.

## Background

The filamentous fungus *Trichoderma reesei* is industrially important fungi used for the production of cellulases and hemicellulases due to its large protein secretion capacity. The secreted cellulase quantities can exceed 100 g/L culture in several industrial strains after rounds of random mutagenesis [1]. With this property, in addition to the production of cellulases, *T. reesei* has also been developed into a host platform to express a variety of recombinant proteins, including both native and heterologous proteins [2, 3]. It is well known that gene expression is strongly affected by its copy number in the genome and the local DNA features close to the integration sites [4, 5]. Indeed, we observed this phenomenon by successful expression of a heterologous lipase gene in *T. reesei* via random insertion of the expression cassette, from which we noticed that the expression levels of lipase varied in different transformants with different insertion site of the *lipA* gene [6]. In *T. reesei*, targeting recombinant genes to the *cbh1* locus is a typical approach to lead to high expression levels and *cbh1* locus is currently the only integration site reported to have positive influence on the expression of recombinant genes [3]. Little is known about whether there are other integration sites in *T. reesei* genome that would be benefit for gene expression, although *T. reesei* genome has been well sequenced and annotated [7, 8].

To search for gene integration sites benefit for gene expression, one of the foremost considerations is to develop a high-throughput, low-cost screen method for screening strains with high expression levels of the recombinant protein from a large population of recombinant strains. Fluorescence-activated cell sorting (FACS) is a specialized application of flow cytometry which is a sensitive and quantitative platform for the measurement of particle fluorescence, and is capable of sorting cells with a special characterization at the single cell level. It has been demonstrated that flow cytometric analysis and fluorescence-activated cell sorting of fungal cells is practicable and this technique has yielded valuable results in a number of different fields of research [9–11]. In *T. reesei*, germinating spores could be used for FACS sorting [12], however, most of the secreted proteins might not be well expressed during the stage of germinating spores. Therefore, using FACS based method to screen *T. reesei* strains bearing relatively high expression of GOI, especially when the GOI is a secreted protein encoding gene, still has lots of challenges. One of the problems is how to manipulate the branched hyphal mats in FACS instruments and how to make the connection between the production yield of extracellular protein and the cell phenotype that can be detected by flow cytometry.

The food-and-mouth disease virus (FMDV) 2A protein is a very small protein that only contains 16–20 amino acids and it is originally responsible for the cleavage of the FMDV poly-protein at its own carboxyl-terminus [13, 14]. When this 2A sequence was inserted between two or more independent genes to form a single ORF transcription unit, upon translation, the constituent proteins could be cleaved apart at the C-terminus of 2A sequence to generate two or more separated gene products [15–17]. With this property, the FMDV 2A peptide has been widely applied in co-expression of two or more genes in a variety of eukaryotic systems [18–20]. Especially the most recently, Subramanian and colleagues reported that heterologous co-expression of a secreted cellobiohydrolase enzyme (Cel7A from *Penicillium funiculosum*) and an intracellular enhanced green fluorescent protein (eGFP) linked by 2A peptide in *T. reesei* resulted in a equal expression ratio of eGFP and Cel7A, indicating that the FMDV 2A peptide is also practicable to co-expression multiple genes in *T. reesei* [21].

In this study, to search for the integration sites that will benefit for gene expression, we employed 2A peptide to co-express a RFP gene and a secreted heterologous lipase gene *lipA* in *T. reesei* and based on which, we established a FACS based high-throughput screening method to screen strains bearing relatively high expression levels of recombinant proteins. Several strains with highly expressed recombinant gene including a strain R5 that the recombinant gene was integrated into *cbh1* locus were successfully screened. Furthermore, we surprisingly found two additional strains R3 and R11 that exhibited comparable expression levels of the recombinant gene as that in R5, while *cbh1* gene was normally expressed. We subsequently investigated the effects of the integration sites in R3 and R11 on the transgene expression. Our results indicated that integration of recombinant genes into the loci identified in R3 and R11 could result in an optimal expression of the targeted genes. Our study herein provided a feasible and advantageous method to efficiently pick out hyper-secretion strains from a large population of strains that bearing random integration of transgene in *T. reesei.* Additionally, the two gene integration sites identified here provided new clues for strain engineering to improve the production of recombinant proteins and other bio-products in *T. reesei*.

## Results

### Construction and expression of 2A self-cleavage peptide linked poly-protein gene in *T. reesei*

**Fig. 1:**
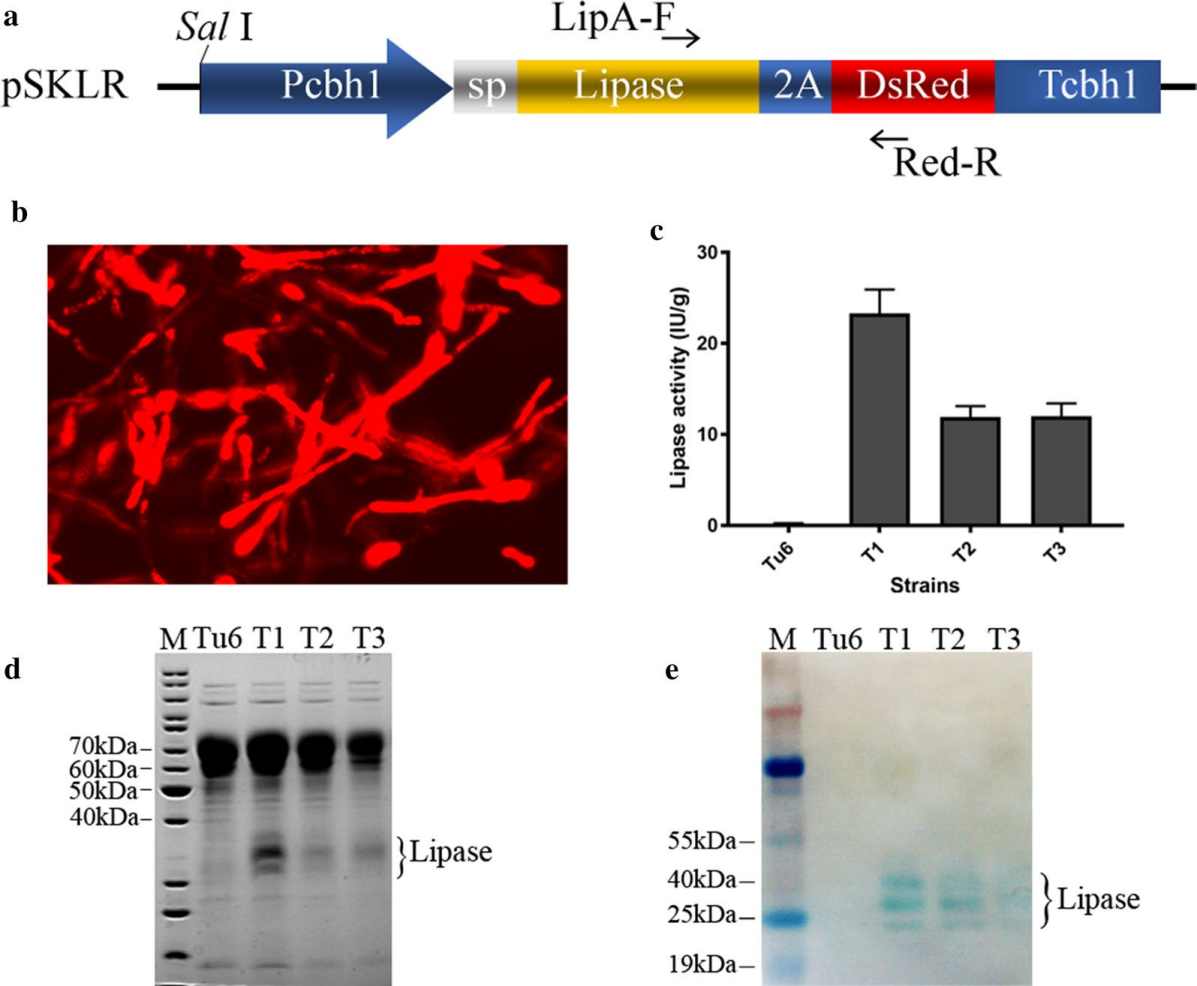
Expression of 2A self-cleavage peptide linked poly-protein gene in *T. reesei.* **a** Schematic diagram of the expression cassette of 2A peptide linked lipase gene and DsRed gene. P*cbh1* represents *cbh1* promoter; T*cbh1* indicates the *cbh1* terminator; sp is the abbreviation of signal peptide; His indicates 6×His tag; 2A indicates 2A peptide; LipA-F and Red-R are primers for genotyping the integration. **b** Representative image showing intracellular expression of RFP which was used to indicate the expression level of extracellular lipase. Images were taken under 40× objective lens using fluorescent microscope-Leica DMI4000B equipped with a mercury lamp. The excitation and emission spectra were 557 and 585 nm, respectively. **c** Lipase activities of three independent transformants and Tu6 strains in the supernatant of 96 h post-inoculation in MM media with 2% of lactose (w/v) and 5 mM uridine. The lipase activity was normalized to IU/g biomass. All the values in the figure are the mean value of three replicates. Error bars are the standard deviation (SD) between these replicates. **d** SDS-PAGE analysis of the supernatant from the same culture as in **c**. **e** Western blot analysis of expressing His-tagged lipA-2A-DsRed fusion protein in *T. reesei*. Samples were as same as that in** d**

To efficiently produce the recombinant lipase in *T. reesei* and use the RFP as an indicator for lipase production yield, the vector plasmid pSKLR including fragments shown in Fig. 1a was designed and constructed. In this construct, the *A. niger lipA* gene and *DsRed1* gene were fused as a polyprotein gene linked by a FMDV 2A self-cleavage peptide. The promoter and terminator of cellobiohydrolase 1 gene (*cbh1*) were used to express the LipA-2A-DsRed expression cassette. The signal peptide sequence of *cbh1* gene was also employed to allow the extracellular secretion of lipase. To facilitate the analysis of the expression of separated lipase and the fused protein using western blot, a His-tag was added in the N-terminus of *lipA* gene. To integrate the pSKLR construct into *T. reesei* chromosome, the linearized plasmid pSKLR and a previous reported plasmid pSK-*pyr4* [6], as a selection marker were co-introduced into *T. reesei* strain Tu6 by PEG mediated protoplast transformation. The transformants were selected on minimal medium (MM) without addition of uridine and confirmed by diagnostic PCR using primer LipA-F and Red-R (Fig. 1a).

Three biologically independent positive transformants were inoculated into MM medium with 2% lactose (w/v) as the carbon source to analyze the expression of the recombinant RFP and lipase. The parental strain Tu6, which is a uridine auxotrophic strain, was used as the negative control. We firstly analyzed the effect of uridine on the protein secretion of strains harboring *pyr4* gene by growing these three transformants with or without the addition of uridine. Our results indicated that uridine did not affect the protein secretion of strains with functional *pyr4* gene (Additional file 1: Figure S1). Therefore, to make the growth conditions consistent, 5 mM uridine was added into the media regardless of whether the strains were uridine auxotrophic strains. After 48 h of induction in lactose, the expressions of RFP in all of these three transformants were observed as red fluorescent mycelium under the fluorescence microscope (Fig. 1b). No red fluorescent was observed in the parental strain Tu6. The extracellular lipase activities in Tu6 and these three transformants were determined in the supernatants of 96 h lactose culture. No lipase activity was detected in Tu6 and the three transformants exhibited obvious lipase activity varied from 10 to 25 IU/g biomass (Fig. 1c). SDS-PAGE analysis showed that compared to Tu6, all of these three transformants had extra lipase bands, further confirming the successful expression of the recombinant lipase (Fig. 1d).

To evaluate the cleavage efficiency of the 2A self-cleavage peptide, western blot analysis was performed using anti-6× His tag antibody to detect the recombinant protein in the supernatant of these three transformants. The data shown in Fig. 1e demonstrated that only three bands were detected in all of the three transformants. Previously, we demonstrated that expression of this *lipA* gene in *T. reesei* generated three separated peptides and MALDI-TOF-TOF mass spectrometry analysis indicated all these peptides bands were *A. niger* lipase [6], which was consistent with the western blot data here, suggesting that no uncleaved protein were detected in the supernatant. To determine whether the uncleaved LipA-2A-DsRed poly-protein failed to secrete and retained in the cell, western blot using the same anti-6× His tag antibody was performed to detect the intracellular proteins from the mycelia lysate. No significant band signal with the right size was detected in all the analyzed transformants (data not shown). These data indicated that the LipA and RFP from the single transcript frame were completely separated after translation using FMDV 2A self-cleavage peptide. Given that the co-expressed proteins via 2A peptide can be theoretically expressed at equal molar ratios, the fluorescent intensity of RFP in each transformant will be a good reporter to estimate the extracellular lipase production yields, which made it possible to use FACS to screen *T. reesei* strains with highly expression of secreted protein of interest.

### FACS screen and quantification analysis of the correlation between red fluorescence intensity and lipase activity

**Fig. 2:**
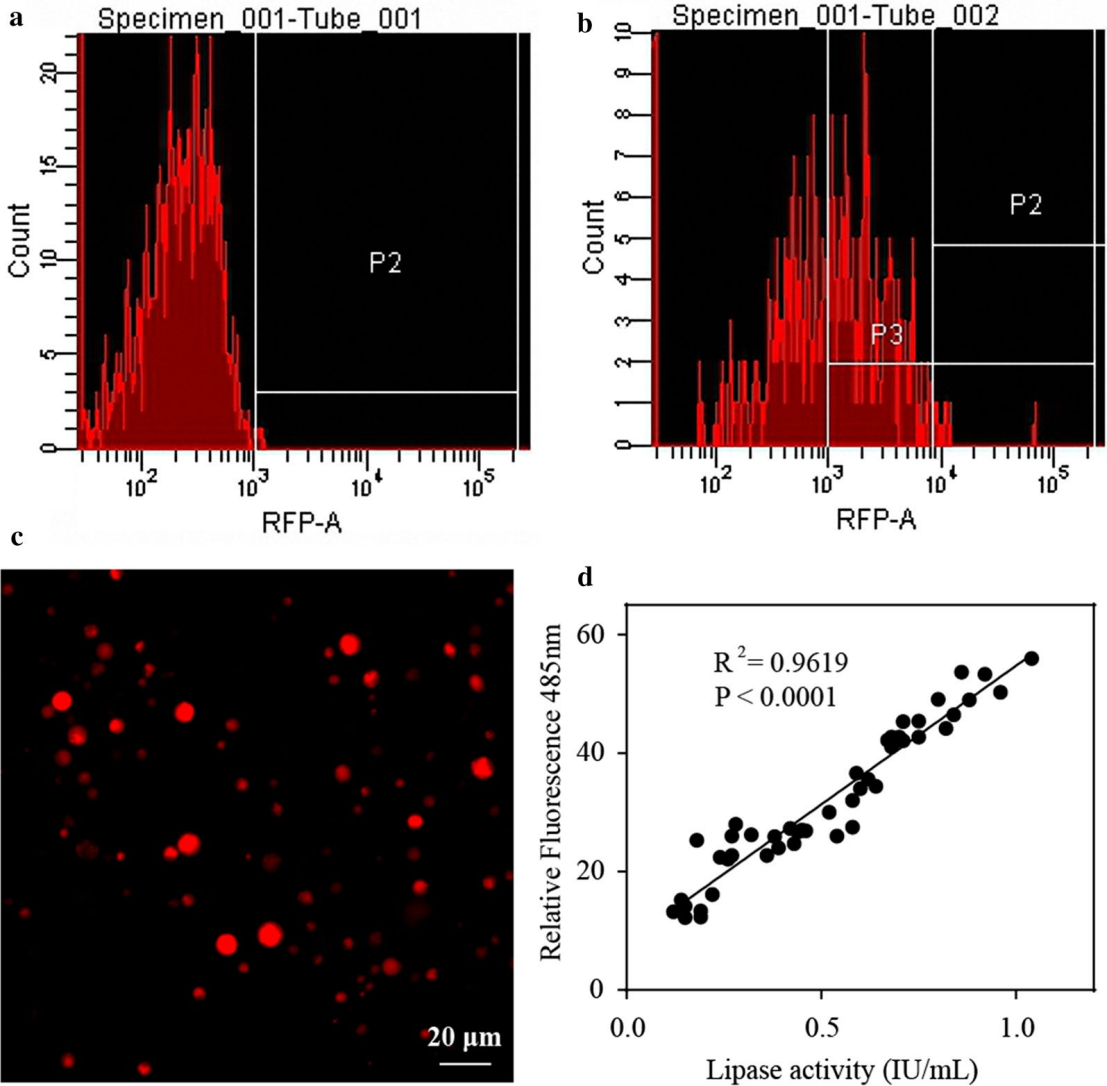
FACS screening and quantification analysis of the expression levels of RFP and lipase. **a**, **b** Representative histographs of flow cytometry analysis of protoplasts from negative control strain Tu6 (**a**) and strains bearing the expression cassette of lipA-2A-DsRed (**b**). Shifted peaks towards right in a histogram represent the increase of fluorescence intensities. **c** Representative image showing that RFP was successfully expressed in the released protoplasts from mycelia. **d** Scatter plots of the fluorescent intensity and lipase activity of the 46 selected recombinant strains. Single protoplasts were sorted into 24 well microtitre plates containing MM media with 0.1% glucose (w/v), 0.1% glycerol (w/v) and 2% lactose (w/v) as carbon source and cultured for 96 h before the indicated measurement. Each data point represented one well of a 24-well plate. Correlation coefficient (*r*) and the *P* value of the correlation were provided

The major challenge of performing flow cytometry based FACS screening in filamentous fungi is that their filament structure makes it hard to go through the stream of fluid for sorting. Even after 24 h growth in lactose, most of the germlings were too large to through the 400 mesh sieve filter. However, at least 48 h induction was required to observe a significant expression of RFP with this construct under lactose. To make the *T. reesei* culture pass through the filter and fit for flow cytometry sorting, a population of protoplast transformant cells were transferred into liquid lactose media including 1 M sorbitol and cultivated for 72 h to regenerate and induce the expression of the recombinant proteins driven by *cbh*-*1* promoter. Mycelium with the obvious expression were collected and lysed to release the protoplasts. From the data shown in Fig. 2a–c, RFP expression was significantly observed in these released protoplasts with different intensity, implying the possibility that the protoplasts suspension could be used for FACS screening. To screen the *T. reesei* strains bearing the relatively higher production of recombinant protein, two rounds of FACS screening were performed. For the first round, protoplasts with 0.03% of the top highest fluorescent intensity were collected together into sorbitol contained lactose media for another 72 h regeneration and induction of the RFP and lipase expression. Protoplasts released from this culture were used to perform the second round of FACS screening. Seventy two single individual protoplasts with the top highest florescent intensity were sorted into 24 microtitre plate wells containing the same media as above (details see “Methods” section).

**Fig. 3:**
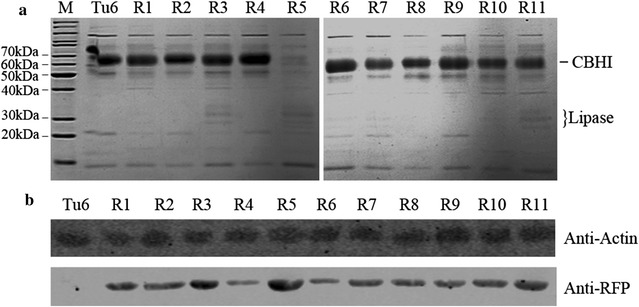
SDS-PAGE and Western blot analysis of the secreted proteins and the intracellular RFP. **a** SDS-PAGE analysis of the supernatant of 11 representative sorted recombinant strains from 120 h cultures containing MM media with 1% Avicel (w/v) as the carbon source from 24 well plates. **b** Western blot analysis of the intracellular RFP expression. Actin protein was used as a reference to normalize the biomass. Protein samples were extracted from mycelia in the same culture as that in **a**. The raw data was shown in Additional file 2: Figure S2

Forty six out of 72 single sorted individual protoplasts were successfully regenerated and their fluorescent intensity and lipase activity were quantitatively measured after 96 h of post-inoculation under lactose media. The Pearson correlation coefficient analysis shown in Fig. 2d indicated that the production of extracellular lipase was well correlated with the fluorescence intensity with a high correlation coefficient (*R*^2^ = 0.9252, 95% confidence interval is 0.9317 to 0.9788, *P* < 0.0001). To further confirm the correlation between the fluorescent intensity and the production level of extracellular lipase, SDS-PAGE analysis was used to detect the expression levels of lipase in the culture supernatants of 11 representative strains (Fig. 3a). In addition, western blot analysis was applied to quantify the expression levels of RFP from the mycelia lysate using the anti-RFP antibody, meanwhile using anti-actin antibody as the reference control (Fig. 3b). The results showed that the expression levels of RFP were highly consistent with the secreted lipase, further confirming that the RFP and recombinant lipase were expressed as an equal ratio. In addition, the data shown in Additional file 2: Figure S2b demonstrated that only one signal band was detected in all of the tested strains, further confirming that the cleavage efficiency of 2A peptide in our constructs was 100%, and lipase could be successfully secreted in a biologically active form while the RFP protein remained in the cell as an indicator for flow cytometric sorting. These data suggested that 2A oligopeptide mediated co-expression of RFP and a secreted protein in *T. reesei* was a feasible method to efficiently screen strains with hyper-secretion of the target proteins using high throughput FACS screening.

### A lipase hyper-secretion strain with cbh1 gene deletion was obtained from FACS screening

**Fig. 4:**
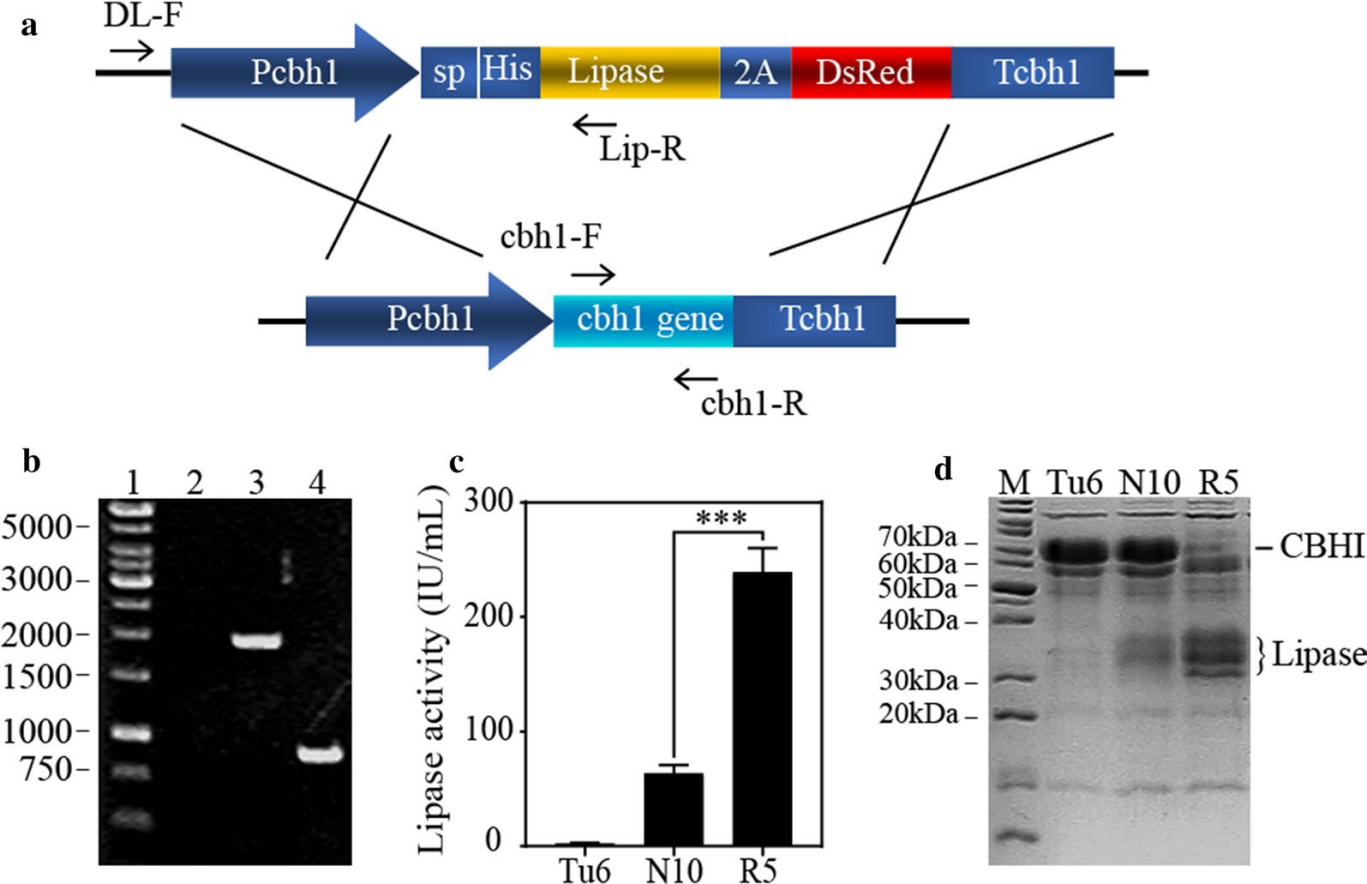
Characterization of the screened strain R5. **a** Schematic diagram of the homologous recombination occurred in strain R5. P*cbh1* represents *cbh1* promoter; T*cbh1* indicates the *cbh1* terminator; sp is the abbreviation of signal peptide; His indicates 6xHis tag; 2A indicates 2A peptide; DL-F, Lip-R, cbh1-F and cbh1-R were indicated primers. **b** Genotyping of *T. reesei* strain R5. Lane1, DNA Marker; Lane2, The result of diagnostic PCR using R5 genomic DNA as the template, primer cbh1-F and cbh1-R as the primer pairs; Lane3, The result of diagnostic PCR using R5 genomic DNA as the template, and primer DL-F and Lip-R as the primer pairs; Lane4, The result of diagnostic PCR using Tu6 genomic DNA as the template, primer cbh1-F and cbh1-R as the primer pairs. **c**, **d** Lipase activities and SDS-PAGE analysis of the strain Tu6, N10 and R5 from 120 h cultures containing MM media with 1% Avicel (w/v) and 1% glycerol (w/v) as the carbon source and 5 mM uridine was added. All the values in the figure are the mean value of three replicates. Error bars are the SD between these replicates

From the data shown in Fig. 3a, we surprisingly found that the large amount of CBHI protein band was disappeared in strain R5. Further genotyping data using diagnostic PCR showed that in strain R5, the expression cassette of Lipase-2A-DsRed was inserted into *cbh1* gene locus via homologous recombination (Fig. 4a, b). We previously reported that knocking down the gene expression level of *cbh1* gene could significantly improve the production level of heterologous lipase. We consequently compared the lipase production level in strain R5 with that in strain N10, a previously constructed strain expressing the same lipase gene with relatively higher level [6]. The data demonstrated in Fig. 4c, d showed that the lipase yield in strain R5 was 3.8-folds higher than that in strain N10. Targeting recombinant genes to the *cbh1* locus to replace the *cbh1* gene has usually been considered as efficient strategy to lead to high expression levels of recombinant proteins [3]. However, the integration of DNA fragment into certain genome locus in *T. reesei* wild type strain is difficult due to the low frequency of homologous integration events. Accordingly it is very hard to get a strain like R5 without an efficient high-throughput screening method. Therefore, the finding of strain R5 provided another solid evidence that the screening method developed here was a reliable strategy to obtain strains containing higher production of secreted proteins of interest.

### Two genome insertion sites benefit for gene expression were identified via plasmid rescue method

From the data shown in Fig. 3a, two strains R3 and R11 exhibited comparative production levels of lipase as that in R5, while *cbh1* gene was normally expressed in these two strains. To figure out the factors associated with the higher expression levels of recombinant lipase in strain R3 and R11, gene copy number and the insertion sites of the transgene were analyzed. Quantitative PCR (qPCR) was employed to identify the copy number of lipase gene in strain R3 and R11 using N10, a strain only contains one copy of lipase gene, as the reference strain [6]. The data shown in Additional file 3: Figure S3a indicated that both R3 and R11 contained only one copy of the expression cassette of Lipase-2A-DsRed. To determine the chromosomal location of the inserted transgene in strain R3 and R11, plasmid rescue strategy was used to capture the fragment including the flanking chromosomal region of the transgene. Total genomic DNA of R3 and R11 were extracted and digested with restriction enzyme *Sal* I, which is the only restriction site of plasmid pSKLR (Fig. 1a), and then religated and transformed into *E. coli* cells. Twenty four colonies for strain R3 and 33 colonies for strain R11 were recovered under selection against ampicillin. Considering that both the transformed plasmids pSK*pyr4* and pSKLR contain ampicillin resistance gene, colony PCR using primers targeting in lipase gene was first performed to get rid of colonies containing the flanking genomic sequence at the pSK*pyr4* insertion site. Eleven out of 24 colonies and 9 out of 33 colonies were verified to contain lipase gene in strain R3 and R11, respectively (Additional file 4: Figure S4a, b).

**Fig. 5:**
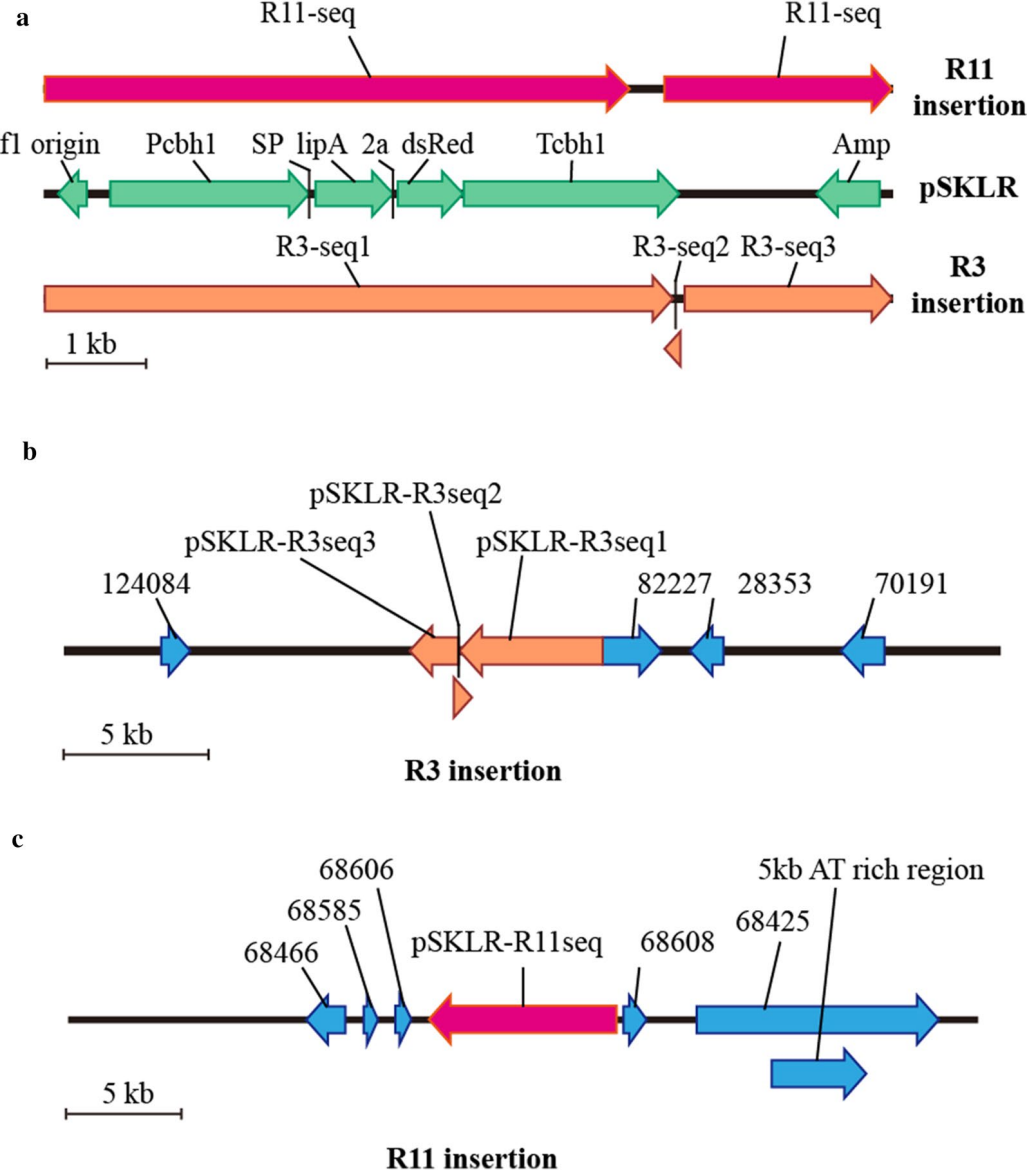
Schematic diagram of pSKLR insertion in strain R3 and R5. **a** Schematic diagram of the plasmid pSKLR. The region highlighted in orange was the rescued sequence from strain R3 and the region highlighted in pink was from strain R11. **b**, **c** Schematic diagram of the integration occurred in R3 (**b**) and R11 (**c**). Numbers labeled in the diagram represented the protein ID of each gene from *T. reesei*

Since the result of qPCR indicated that there was only one copy of Lipase-2A-DsRed expression cassette in the genome of R3 and R11, theoretically the sequencing results of all of recovering bacterial plasmids from the same *T. reesei* strain should be identical. We then selected three recovering bacterial plasmids, respectively, from the strain R3 and R11 for sequencing and performed diagnostic PCR to determine if all of recovered plasmids were identical. As expected, the sequencing results of the three plasmids from both R3 and R11 strain were exactly same and the results of diagnostic PCR indicated that all of the plasmids recovered from the same *T. reesei* included same genomic flanking sequence (Additional file 4: Figure S4c, d), further confirming that there was only one copy of the lipase gene in the genome of R3 and R11. The sequencing results showed that most of pSKLR sequence was included in the recovered plasmids from both R3 and R11 genome. There was inverse insertion of 60 bp (6548–6607 bp in pSKLR) occurred and the first two base pairs were missing in R3 insertion (Fig. 5a, b, Additional file 5), while for R11 insertion, 355 bp (6101–6455 bp in pSKLR) were missing (Fig. 5a, c, Additional file 5). There were 337 and 248 bp of flanking genomic sequence of the R3 and R11 insertion were, respectively, recovered (Additional file 5). A search of the *T. reesei* genome on the basis of the *T. reesei* genome database v.2.0 (http://www.genome.jgipsf.org/Trire2/Trire2.home.html) using these two flanking sequences as queries showed that both sequence only had one hit against the *T. reesei* v2.0 filtered model transcripts database and the *T. reesei* v2.0 assembly database, respectively. The pSKLR construct integrated into the 5′UTR region of *cel3c* gene (protein ID 82227), which was predicted to encode a intracellular ß-glucosidase [22] in strain R3, while in strain R11, the pSKLR construct was inserted into the non-coding region between a coding gene predicted to encode triosephosphate isomerase (protein ID 68606) and the gene *thi4* predicted to encode thiazole biosynthetic enzyme (protein ID 68608). The detail information about these two insertion sites was shown in Fig. 5 and Additional file 5.

To determine whether the integration sites identified in this study were conserved in other *Trichoderma* species, we performed sequence alignments using 4 kb sequence surrounding the R3 and R11 insertion sites as queries to against 9 sequenced *Trichoderma* species including *T. reesei* Rut C-30, *T. asperellum* CBS 433.97*, T. asperellum TR356, T. citrinoviride* TUCIM 6016, *T. gamsii* T6085, *T. harzianum* CBS 226.95, *T. harzianum* TR274, *T. longibrachiatum* ATCC 18648 and *T. virens*. Gv29-8 (Additional file 6: Table S1). All of these genome sequences are available in JGI Genome Portal (https://genome.jgi.doe.gov/portal/). The results showed that *T. reesei* species RutC30 and QM6a shared 100% identity and 100% coverage with both 4 kb R3 and R11 loci surrounding sequences, while *T. citrinoviride* shared 98% coverage and 81.59% identity with R3 locus sequence, 87% coverage and 89.97% identity with R11 sequences, and *T. longibrachiatum* shared 75% coverage and 85.57% identity with R3 locus sequence, 88% coverage and 89.23% identity with R11 sequences. However, other *Trichoderma* species had a significantly lower coverage ratio with both R3 and R11 locus sequences (Additional file 6: Table S2), implying that the identified locus may be only applied for a few *Trichoderma* species.

### Functional analysis of the above two transgene insertion sites

In general, the copy number and the integration site of an introduced transgene are highly related to the ultimate expression level of the target gene [4, 5, 23]. Since in both R3 and R11 strain, there was only one copy number of the Lipase-2A-DsRed cassette in the genome, the integration sites identified in R3 and R11 may play a key role in their higher expression level of the recombinant gene. In *T. reesei*, *cbh1* gene was one of the highly expressed endogenous genes and targeting transgenes into *cbh1* site was one of the efficient strategies to improve the expression levels of the indicated genes [3]. Our data demonstrated that the production levels of lipase in R3 and R11 were comparable to that in strain R5, implying that the integration sites in R3 (R3 locus) and R11 (R11 locus) might have the same or even better effect on gene expression compared to *cbh1* locus. To verify this hypothesis, we constructed strains, in which *cbh1* gene was integrated into R3 locus, R11 locus, or other random sites in the genome, respectively. Meanwhile, *cbh1* gene in its native locus was replaced by *lipA* gene. These generated strains were, respectively, named as R3*cbh1*, R11*cbh1* and R*cbh1* accordingly. To improve the homologous recombination ratio, the strain *Tu6∆ku70* [24] was used as parental strain to generate R3*cbh1* and R11*cbh1* strains and Tu6 was used to generate R*cbh1* series strains. The copy number of *cbh1* gene in these transformants was first determined using qPCR. The data shown in Additional file 3: Figure S3b only listed the resulted strains, which contains single copy number of *cbh1*gene.

**Fig. 6:**
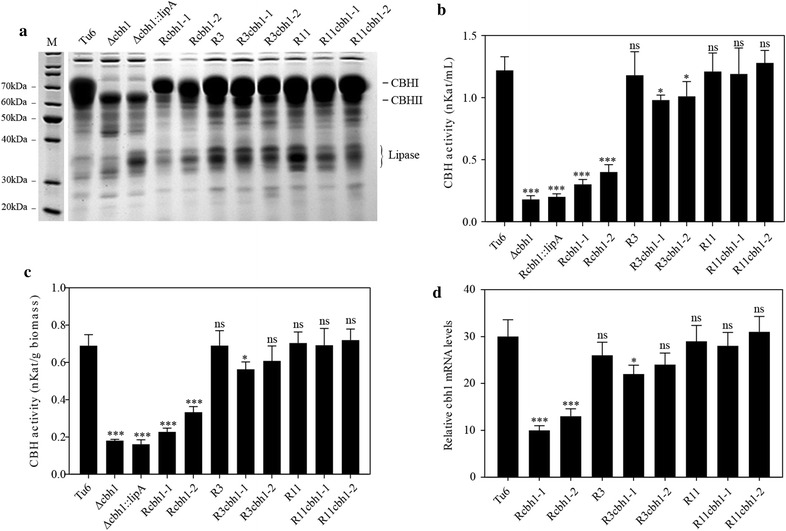
Effects of the R3 and R11 loci on the expression levels of *cbh1* gene. **a** SDS-PAGE analysis of the supernatants of the indicated strains cultured in 144 h of MM media containing 1% Avicel (w/v) as the carbon source. **b** Measurement of the total cellobiohydrolase activities of the indicated strains as the same condition as in **a**. **c** Measurement of the total cellobiohydrolase activities of the indicated strains cultured in 120 h of MM media containing 2% lactose (w/v) as the carbon source and normalized the activity by biomass. **d** qRT-PCR analysis of the expression levels of *cbh1*gene. RNA was extracted from the mycelia of 96 h culture in MM media with 1% Avicel (w/v) as carbon source. All the values in the figure are the mean value of three replicates. Error bars are the SD between these replicates. Asterisks indicate significant differences (*P < 0.05; **P < 0.01; ***P < 0.001). *ns* not significant

To evaluate the expression levels of *cbh1* gene in these recombinant strains, two R*cbh1* strains R*cbh1*-*1* and R*cbh1*-*2*; two R3*cbh1* strains R3*cbh1*-*1* and R3*cbh1*-*2*; and two R11*cbh1* strains R11*cbh1*-*1* and R11*cbh1*-*2*; along with the control strains Tu6, ∆*cbh1*, ∆*cbh1::lipA*, R3 and R11 were inoculated into the MM media with 1% Avicel as carbon source at the conidia concentration of 2x10^6^/mL and cultivated for 144 h. SDS-PAGE analysis of the supernatant of each culture showed that compared to Tu6 and R3, the secreted CBHI protein level in strains R3*cbh1*-*1* and R3 *cbh1*-*2* were slightly lower, while the CBHI protein levels in the two R11*cbh1* strains were comparable to that in R11 and Tu6. The strains with random insertion of *cbh1* gene and with *cbh1* deletion showed significantly decreased levels of CBHI expression (Fig. 6a). To further confirm the results observed from SDS-PAGE analysis, the activity of cellobiohydrolase (CBH) was measured using 4-methylumbelliferyl-ß-d-cellobiose as the substrate. The data shown in Fig. 6b demonstrated that consistent with the SDS-PAGE results, the CBH activities in R3*cbh1* strains were lower than that in Tu6 and R3, and the R11*cbh1* strains displayed a similar CBHI activity as that in Tu6 and R11, while CBHI activity in other tested strains were significantly lower (Fig. 6b). To analyze the effect of different biomass accumulation on the secretion of CBHI, we inoculated these strains into MM media with 2% lactose (w/v) as the sole carbon source and normalized the CBH activity by per biomass. The results showed that CBH activity trends in these tested strains were similar as that under Avicel condition (Fig. 6c), indicating that the different expression levels of *cbh1* gene in these strains were not caused by the different biomass accumulation. These data indicated that the high level of *cbh1* gene expression in *T. reesei* was position dependent and the locus identified in R11 had comparable effect on gene expression as *cbh1* locus. Although the locus identified in R3 was not as good as *cbh1* locus for gene expression, compared to other random sites, it still was an alternative gene insertion site that more possible to result in higher expression of target genes.

It has been reported that the chromosome location of a trangene could affect its expression at the level of transcription [23, 25]. To investigate whether the effect of insertion sites on gene expression occurred at the transcriptional level or post-transcriptional level, qRT-PCR was performed in strain Tu6, R*cbh1*-*1, Rcbh1*-*2*, R3, R3*cbh1*-*1,* R3*cbh1*-*2, R11, R11cbh1*-*1* and *R11cbh1*-*2* to detect the transcriptional levels of *cbh1* gene. The data exhibited in Fig. 6d showed that the expression pattern of *cbh1* gene in these strains at the transcriptional level was highly consistent with their protein expression pattern, suggesting that the position effects on gene expression in these concerned strains from the present study occurred at the transcriptional level.

## Discussion

Although the market share of *T. reesei* based cellulases and hemi-cellulases were diminishing in recent years, the application of using *T. reesei* as a cell factory to produce other valuable protein products still has great potential due to its large capacity of protein secretion. For construction of host strain to express recombinant proteins, gene copy number and the selected promoter are usually considered as the major factors to affect the transgene expression. However, the expression levels of the transgenes are quite different when placed in different chromosomal locations. This phenomenon has been reported in several systems such as *Escherichia coli* [26], *Drosophila melanogaster* [5], human cells [27], *S. cerevisiae* [28] as well as *T. reesei* [6]. Considering that chromosomal integration was required for the construction of recombinant *T. reesei* strains to express homologous or heterologous genes, in addition to the gene copy number and the promoter, epigenetic effects in surrounding of the chromosomal integration site should also be an important consideration when constructing recombinant *T. reesei* strains.

Compared to the random integration, site-specific integration has lots of advantages such as the ability to direct transgenes to a neutral location to avoid insertion mutagenesis. However, since no systematic studies have been made to identify transgene integration loci that enable an optimal gene expression in *T. reesei*, site-specific integration could only be a downstream step of the random integration, due to the uncertainties about which loci could lead to the optimal transgene expression. Currently, the most commonly used site for targeted integration has been *cbh1,* a locus that harbors the expression of the main endogenous secreted protein CBHI in *T. reesei*. Integration of transgene into *cbh1* locus to replace *cbh1* gene has been proved to be an efficient strategy to result in sufficiently high levels of transgene expression [29]. However, the strong *cbh1* promoter was usually used in this strategy and suppression of *cbh1* gene also can contribute to improve the expression level of target gene [6], which made it hard to determine whether the high expression levels were caused by the integration position effect. Furthermore, deletion of *cbh1* gene can cause growth defect on cellulose based carbon sources, because of the decreased efficiency of releasing cellobiose from cellulose. Therefore, searching for additional favorable integration sites in *T. reesei* for secreted expression of transgenes will be especially important.

For the above purpose, we employed the FMDV 2A peptide to co-expression of a secreted heterologous lipase gene *lipA* and RFP protein in *T. reesei*. In this system, the fluorescent intensity of the intracellular RFP can be used as a good reporter to indicate the production levels of the secreted lipase. We then used this system to successfully develop a FACS based high throughput screening method to screen strains with optimal transgene expression, from a pool of recombinant strains bearing random integration. Among the 46 screened strains, strain R5, R3, and R11 caught our attention to perform further investigation. Our results showed that R5 was a strain that the transgene was inserted into *cbh1* site and the native *cbh1* gene was replaced, while R3 and R11 displayed similar expression levels of transgene without a deletion of *cbh1* gene (Fig. 3a). The single copy number of transgene in strain R3 and R11 indicated that the integration site in these two strains might play a major role in the high transgene expression levels. The results of the recombinant expression of endogenous *cbh1* gene in the newly identified R3 and R11 locus confirmed this hypothesis (Fig. 6). Our study here provided a promising screening method to screen strains with higher secreted expression of transgene and with this approach, we further revealed two previously unrecognized loci that enable transgenes be reliably expressed at high levels by integration into a single locus. However, these two genome loci were only conserved in a few *Trichoderma* species according to the sequences alignment data shown in Additional file 6: Table S2. In addition, one of the limitations of the screening method we developed here is the low transformation efficiency of *T. reesei.* The efficiency of the commonly used PEG mediated protoplast transformation was only 200–300 colonies per microgram plasmid DNA when using *pyr4* as the selected marker [12], which is far from to cover all of the genome insertion loci. Hence, establishment of a more efficient transformation system will contribute to identify more integration sites that benefit for the transgene expression.

Integration position effects on transgene expression levels have been reported to associate with chromatin structure [30]. For example, transcriptional silencing could be caused if the transgenes located in telomeric region [31], while higher transcriptional level could be obtained when transgenes were close to the DNA replication origins [28]. Our results showed that the high expression of transgenes occurred at the transcriptional level in both R3 and R11 integration (Fig. 6d). Further analysis of the sequence around R11 locus revealed that there were about 5 kb AT rich sequences in the downstream of insertion site (Fig. 5c and Additional file 5), which is a part of the intron region of a predicted fungal specific transcription factor (protein ID 68425) encoding gene. It has been well known that the high AT content sequence is a common character for DNA replication initiation in bacterial, archaeal, and eukaryotic replicons [32]. In yeast, AT-rich DNA sequence can contribute to remove the nucleosomes by the RSC chromatin remodeling complex to form the nucleosome-free regions (NFRs) [33]. Considering that NFRs were associated with the initiation of transcription of most genes, the high transgene expression levels in R11 locus might be benefited from the 5 kb AT-rich sequence in the downstream of the insertion site, although little studies associated the function of AT-rich DNA sequence was performed in *T. reesei* to date.

For R3 locus, the recombinant gene was integrated into the 5′UTR region of the *cel3c* gene. No specific characterized sequence was found around the R3 locus. However, integration occurred in R3 locus might disrupt the expression of *cel3c* gene, which predicted to be a β-glucosidase encoding gene [22]. Recently, it has been reported that dysfunction of a β-glucosidase encoding gene *cel3d* in *T. reesei* resulted in higher secretion of cellulases [34]. In addition, in *N. crassa,* deletion of β-glucosidases could efficiently decrease the carbon catabolite repression (CCR) effect, thereby allowing the induction of cellulases under cellobiose, cellotriose and cellotetraose [35]. Considering that decreased CCR could result in higher expression of cellulose-induced proteins [29], we presumed that the high expression level in R3 locus might result from the disruption of *cel3c* gene. To verify this hypothesis, we made a *cel3c* deletion strain and compared the total secreted protein levels and cellulase activity between ∆*cel3c* and its parental strain. However, unexpectedly, our data demonstrated that the deletion of *cel3c* gene had no significant influence on the expression of secreted proteins (data not shown). We then checked the upstream region of the *cel3c* gene and found the promoter region of *cel3c* has been predicted to have putative XYR1-binding site [36]. It is well known that the transcriptional factor *xyr1* is the major activator for most cellulases genes, which might contribute to the high expression level of the targeted gene. However, the mechanism behind the R3 and R11 position effect on the transgene expression still needs to be further investigated.

## Conclusions

The chromosomal location of transgenes plays an important role in the ultimate recombinant protein yield. To search and identify loci for optimal transgene expression in *T. reesei*, we employed 2A mediated multiple proteins co-expression system to simultaneously express a secreted lipase gene and the RFP gene, by randomly integration of the expression cassette into *T. reesei* genome. Our data demonstrated that in this system, the production levels of the extracellular lipase were well correlated with the intracellular RFP fluorescent intensity, thereby allowing us to perform flow cytometry sorting for screening strains with better secretion yields of the transgene (in this case, *lipA* gene). We subsequently investigated the copy number and the integrated sites among the screened strains and eventually identified two optimal loci for transgene expression in *T. reesei*. In the short term our study here provided a promising strategy to construct optimal *T. reesei* host strains for the production of recombinant proteins, and in the longer term, it raised the question how these two loci eventually affected the gene expression. Further investigation of the behind mechanism will contribute to a biological understanding of the epigenetic effects on transgene expression.

## Methods

### Microbial strains and growth conditions

*Trichoderma reesei* Tu6 strain (ATCC MYA-256) [37] was obtained from American Type Culture Collection (ATCC). *T. reesei Tu6∆ku70* [24] was kindly provided by Prof. Dr. Monika Schmoll (AIT Austrian Institute of Technology, Austria). *T. reesei* strain R3, R5, R11 were constructed by co-transforming strain Tu6 with plasmid pSK-pyr4 [6] and pSKLR. The plasmid of pSKLR was derived from plasmid pBluescript SK (+) by inserting a fused DNA fragment containing the dsRed1 gene, 2A sequence and a heterologous lipase gene lipA under the control of the *cbh1* promoter (P*cbh1*) and *cbh1* terminator (T*cbh1*) from *T. reesei* (Fig. 1a). Transformants were selected on MM media [38] without adding uridine and verified by diagnostic PCR.

The R3*cbh1* and R11*cbh1* series strains were generated by co-transforming strain Tu6∆*ku70* with three DNA fragments including a linearized pSK-*pyr4*, a fragment containing the expression cassette of P*cbh1*-*lipA*-T*cbh1* and a fragment including the expression cassette of P*cbh1*-*cbh1*-T*cbh2* flanked with 2 kb upstream and downstream DNA sequence of R3 or R11 locus. ∆*cbh1*::*lipA* strains were generated by only co-transforming strain Tu6∆*ku70* with two DNA fragments including a linearized pSK-*pyr4* and a fragment containing the expression cassette of P*cbh1*-*lipA*-T*cbh1.* Transformants were selected on minimal media without adding uridine and tested for genotypes by diagnostic PCR. The R*cbh1* series strains were created by co-transforming strain Tu6 with linearized plasmid pSK-*pyr4* and a fragment including the expression cassette P*cbh1*-*cbh1*-T*cbh2*. Transformants were selected on minimal media without the addition of uridine and verified by diagnostic PCR. Strain ∆*cbh1* was generated by transforming strain Tu6∆*ku70* with a DNA fragment that contained *pyr4* expression cassette flanked with 2 kb upstream and downstream DNA sequence of *cbh1* gene. Transformants were selected on minimal media without uridine and verified by diagnostic PCR. All the primers used in this study were listed in Additional file 7: Table S3.

For conidiation*, T. reesei* strains were grown for 5–6 days at 28 °C on potato dextrose agar plates (PDA) or PDA supplemented with 5 mM uridine when necessary. For measurement of the secreted proteins, 2 × 10^6^ conidia/mL were inoculated into 50 mL of liquid minimal medium with the indicated carbon source in 250 mL flasks and grown at 28 °C on a rotary shaker (200 rpm) in continuous dark condition for the indicated time. 5 mM uridine was added when using Tu6 or *Tu6∆ku70* as a control to compare the protein secretion change in other recombinant strains, which derived from these two strains. 1% glycerol (w/v) was added when using strains with *cbh1* gene deletion background.

### Sphere-protoplasting and transformation of *T. reesei*

The indicated *T. reesei* strains were inoculated into slants and cultivated for 5–7 days for conidiation. The conidia were suspended by adding 3 mL sterile H_2_O into the slants and filtered through a metal sieve of 200-mesh. Then the filtered conidia were inoculated into 100 mL minimal medium with 2% glucose as carbon source at 2× 10^6^/mL and grown at 28 °C on a rotary shaker (220 rpm) for 13–14 h. The germinated mycelium from this culture was collected by filtering through a 200-mesh sieve and re-suspended into 15 mL of 0.2 M phosphate buffer, pH7.4, which contained 150 mg lysing enzyme (Sigma, L1412) and 15 mg cellulase (Onozuka, R-10), and incubated at 30 °C on a rotary shaker (80 rpm) for 1.5–2 h. The culture was added with equal volume of 0.6 M sorbitol solution including 0.6 M sorbitol and10 mM Tris–HCl, pH7.0 and filtered using 200-mesh sieve to remove the undigested mycelia. The flowthrough including protoplasts were centrifuged at room temperature at 2800 rpm for 6–8 min to precipitate the protoplasts. The protoplasts were washed twice using 1.2 M sorbitol solution containing 1.2 M sorbitol, 50 mM CaCl2 and 10 mM Tris–HCl, pH7.4 and re-suspended in 200 μL of the same solution.

For transformation, the above protoplasts suspension was mixed with 5–10 μg transformed DNA (the total DNA volume should be ≤ 20 μL) and 50 μL PEG solution including 50% PEG4000, 50 mM CaCl_2_ and 10 mM Tris–HCl, pH7.4. The mixture was incubated on ice for 30 min and then added with 1 mL PEG solution and incubated at room temperature for 20 min. 1 mL sorbitol was added after PEG treatment. For normal transformation, the mixture was added into MM media with 0.8% agar and spread onto selective plates containing 1 M sorbitol and the plates were incubated at 28 °C for 3–5 days [6]. For flow cytometry screening, the protoplasts preparation and transformation were performed as described above, excepting that the last step was changed (see the details below).

### Flow cytometry and FACS screening

The construct pSKLR was co-transformed into the *T. reesei* strain Tu6 (an auxotrophic strain) with the plasmid pSK*pyr4*, which contains the *pyr4* gene that complements uridine auxotrophic stains. For regeneration, the transformed protoplast suspension was transferred into 50 mL minimal medium as described previously [38], except that the carbon source 2% glucose (w/v) was substituted with 0.1% glucose (w/v), 0.1% glycerol (w/v) and 2% lactose (w/v), in addition, 1 M sorbitol was included as osmotic stabilizer.

For flow cytometry analysis, after 72 h incubation, all of the regenerated mycelium above was collected for another protoplast preparation. Protoplast suspensions were filtered through a metal sieve of 400-mesh and cytometrically analyzed and sorted with a FACSAria (BD Biosciences) using phosphate buffered saline as a sheath fluid. The sheath pressure was set at 70 psi, and the defection plate voltage was set at 5000 V (default ‘‘low’’ setting). A 488-nm coherent sapphire solid state laser was used for excitation, and emission was measured at 576/26 nm. The photomultiplier tube voltage was set at 330 V for forward scatter, 330 V for side scatter, and 650 V for RFP. The threshold value for event detection was set at 5000 on forward scattering. The drop drive frequency was set to approximately 87 kHz, and the amplitude was set to approximately 33 V; the drop delay value was approximately 44.78.

Protoplasts with the highest fluorescence value (top 0.03%) were directly sorted into 1 M sorbitol included minimal medium with 0.1% glucose (w/v), 0.1% glycerol (w/v) and 2% lactose (w/v) as carbon source and incubated 72 h at 28 °C. Protoplast preparation and sorting procedure were repeated. In the second round of sorting, single protoplasts were sorted into individual wells of 24 well plates containing the same medium as above.

### Measurement of in vivo RFP fluorescence in *T. reesei* mycelium

The sorted protoplasts in three 24-well plates were incubated for 96 h at 600 rpm at 28 °C in a microplate shaker (Multitron II. Infors HT). The mycelia from each well were then filtered through 200 mesh filter (30 µm pore diameter) and added 500 µL Tris–HCl (pH7.5) and lysed using a mini-bead beater (Biospec Products, Bartlesville, Okla.) with 0.5 mm diameter glass beads. The mixture was centrifuged for 5 min at 12,000 rpm and the supernatant was carefully removed for analysis. The RFP fluorescent was measured using a Synergy H4 Hybrid Microplate Reader with 557 nm as the excitation wavelength and 585 nm as the emission wavelength.

### Enzyme activity assay of lipase and cellobiohydrolase

Lipase activity was quantitatively determined by an alkali titration method [39] using olive oil as the substrate when using the supernatant of *T. reesei* culture from flasks. The reaction was carried out in 50 mM Tris–HCl buffer, pH 7.5 for 10 min at 45 °C. One unit of lipase activity was defined as the amount of lipase necessary to liberate 1 µmol fatty acid from olive oil per min under the standard assay conditions. Lipase activity was assayed by the colorimetric method using 4-nitrophenyl palmitate as substrate when the supernatant of *T. reesei* culture from 24 well plates was used for analysis. The assay was performed as described by Kumar [40], except that the reaction temperature was 40 °C. One unit (IU) of lipase activity was defined as the amount of enzyme that liberates 1 µmol 4-nitrophenol per minute under assay conditions. Cellobiohydrolase activity was measured with soluble 4-methylumbelliferyl-ß-d-cellobiose (Sigma) as the substrate as previously described [41].

### Western blot

Total proteins (about 50–100 µg) from the supernatant of 96 h shake flasks in minimal medium with 2% lactose (w/v) as the carbon source were separated by SDS-PAGE on 12.5% polyacrylamide gel. Proteins were blotted onto PVDF membrane using Trans-Blot Electrophoretic Transfer (BioRad). The membrane was treated with a diluted (1:1000) anti-His antibody (Tiangen, China) and detected with BCIP/NBT detection kit (CWBIO, China) according to the manufacturer’s introduction. For the western blot analysis of RFP and beta-actin, intracellular proteins were extracted by grinding the frozen mycelia to a fine powder and adding with the HEPES lysis buffer including 50 mM HEPES pH 7.5, 5 mM EDTA pH 8.0, 2 mM EGTA pH 8.0, 100 mM NaCl, 1% Triton X (v/v) and 10% glycerol (w/v). The mixture was centrifuged at 12,000 rpm for 10 min at 4 °C. The supernatant was used to perform western blot using anti-RFP antibody and anti-beta-actin antibody (YESEN, China).

### Plasmid rescue method

Genomic DNA was extracted from strain R3 and R11 as follows: 100 mg mycelium was break down in 400 μL lysis buffer (50 mM sodium phosphate at pH7.4, 1 mM EDTA and 5% glycerol) with 0.3 g silica beads (0.5 mm) using Bead Beater (BioSpec, USA). The lysis was incubated at 65 °C for 30 min and thereafter added with 80 μL Tris–HCl, pH 7.5. The supernatant was transferred into another tube and added with an equal volume of phenol:chloroform and centrifuged. DNA pellet was precipitated by 2 volume of 100% ethanol and washed by 75% ethanol (v/v) and dissolved into 20 μL ddH_2_O.

10 μg of gDNA was digested with *Sal* I at 37 °C for overnight. The digested DNA was precipitated with 3 M sodium acetate, pH 5.2 and ethanol and dissolved in 50 µL ddH_2_O. T4 DNA ligase was used for cyclizing the digested DNA and the reactions were incubated overnight at 16 °C and thereafter precipitated with 3 M sodium acetate, pH 5.2 and ethanol. Samples were dissolved in 10 µL of ddH_2_O and 1 µg of ligated DNA was transformed into *Escherichia coli* strain DH5α competent cells (TransGene Biotech, China) and colonies were selected for ampicillin resistance. The plasmids included in these colonies were sequenced to capture the flanking genomic sequence at insertion sites.

### Gene copy number analysis by qPCR

Genome DNA of the tested strains was extracted as described as above. The DNA concentration and purity was analysed by spectrophotometry (NanoDrop 2000C, Thermo Scientific) and thereafter diluted to the concentration of 20 ng/μL for qPCR reaction. qPCRs were performed in an ABI 7000 real-time detection system using a TransStart Green qPCR SuperMix (TransGene Biotech, China). Total reaction volume for each sample was 20 μL and contained 20 ng of genomic DNA, 10 pmol of each primer, 10 μL of SYBR Green I master mix (TransStart Green qPCR SuperMix, TransGene Biotech, China) and PCR condition was used as the default protocol recommended by the manufacturer. All PCRs were carried out in triplicates within a plate, and two different plates were set up for the same samples. Analysis of the expression level was done using actin gene as a reference. The Ct value of each test gene was calculated by subtraction of reference gene Ct value from each test gene Ct value (Ct = Ct_test_ − Ct_actin_). A control strain, which was known to only contain one copy number of the test gene, was used as reference strain to quantify the gene copy number of the test strains by the 2^−ΔΔCT^ method [42].

### Statistical significance tests

Statistical significance was determined by *t* test analysis by the false discovery rate (FDR) approach using the Prism GraphPad software. Asterisks indicate significant differences (* *P* < 0.05; ** *P* < 0.01; *** *P* < 0.001). ns, not significant.

## Additional files

**Additional file 1.** Analysis of the effect of uridine on protein secretion in strains harboring *pyr4* gene.

**Additional file 2.** The raw data of the western blot analysis of the intracellular expression of RFP and beta-actin from the mycelia lysate.

**Additional file 3.** qPCR to determine the copy number of *lipA* gene and *cbh1* gene in the indicated strains.

**Additional file 4.** Genotyping of the recovered plasmids by diagnostic PCR.

**Additional file 5.** Sequence information of the integration of pSKLR in strain R3 and R11.

**Additional file 6.** Analysis of the conservation of the identified R3 and R11 loci in other Trichoderma species.

**Additional file 7.** Primers used in this study.

## Abbreviations

GOI: gene of interest
RFP: red fluorescent protein
eGFP: enhanced green fluorescent protein
CBHI: cellobiohydrolase I gene
FACS: fluorescence-activated cell sorting
FMDV: the food-and-mouth disease virus
MM: minimal medium
ARS: autonomously replicating sequences
ATCC: American type culture collection
PVDF: polyvinylidene difluoride
SD: standard deviation

## References

[1] Cherry JR, Fidantsef AL (2003). Directed evolution of industrial enzymes: an update. Curr Opin Biotechnol.

[2] Bischof RH, Ramoni J, Seiboth B (2016). Cellulases and beyond: the first 70 years of the enzyme producer *Trichoderma reesei*. Microb Cell Fact.

[3] Singh A, Taylor LE, Vander Wall TA, Linger J, Himmel ME, Podkaminer K, Adney WS, Decker SR (2015). Heterologous protein expression in *Hypocrea jecorina*: a historical perspective and new developments. Biotechnol Adv.

[4] Wilson C, Bellen HJ, Gehring WJ (1990). Position effects on eukaryotic gene expression. Annu Rev Cell Biol.

[5] Markstein M, Pitsouli C, Villalta C, Celniker SE, Perrimon N (2008). Exploiting position effects and the gypsy retrovirus insulator to engineer precisely expressed transgenes. Nat Genet.

[6] Qin LN, Cai FR, Dong XR, Huang ZB, Tao Y, Huang JZ, Dong ZY (2012). Improved production of heterologous lipase in *Trichoderma reesei* by RNAi mediated gene silencing of an endogenic highly expressed gene. Bioresour Technol.

[7] Martinez D, Berka RM, Henrissat B, Saloheimo M, Arvas M, Baker SE, Chapman J, Chertkov O, Coutinho PM, Cullen D (2008). Genome sequencing and analysis of the biomass-degrading fungus *Trichoderma reesei* (syn. *Hypocrea jecorina*). Nat Biotechnol.

[8] Li WC, Huang CH, Chen CL, Chuang YC, Tung SY, Wang TF (2017). *Trichoderma reesei* complete genome sequence, repeat-induced point mutation, and partitioning of CAZyme gene clusters. Biotechnol Biofuels.

[9] Blum A, Benfield AH, Stiller J, Kazan K, Batley J, Gardiner DM (2016). High-throughput FACS-based mutant screen identifies a gain-of-function allele of the *Fusarium graminearum* adenylyl cyclase causing deoxynivalenol over-production. Fungal Genet Biol.

[10] Loll-Krippleber R, Feri A, Nguyen M, Maufrais C, Yansouni J, d’Enfert C, Legrand M (2015). A FACS-optimized screen identifies regulators of genome stability in *Candida albicans*. Eukaryot Cell.

[11] Throndset W, Kim S, Bower B, Lantz S, Kelemen B, Pepsin M, Chow N, Mitchinson C, Ward M (2010). Flow cytometric sorting of the filamentous fungus *Trichoderma reesei* for improved strains. Enzyme Microb Technol.

[12] Hao L, T-h WANG, Y-k ZHANG (2008). Isolation of *Trichoderma reesei* pyrG negative mutant by UV mutagenesis and its application in transformation. Chem Res Chin U.

[13] Ryan MD, Drew J (1994). Foot-and-mouth disease virus 2A oligopeptide mediated cleavage of an artificial polyprotein. EMBO J.

[14] Donnelly ML, Hughes LE, Luke G, Mendoza H, ten Dam E, Gani D, Ryan MD (2001). The ‘cleavage’ activities of foot-and-mouth disease virus 2A site-directed mutants and naturally occurring ‘2A-like’ sequences. J Gen Virol.

[15] Ma C, Mitra A (2002). Expressing multiple genes in a single open reading frame with the 2A region of foot-and-mouth disease virus as a linker. Mol Breeding.

[16] de Felipe P (2003). Co-translational, intraribosomal cleavage of polypeptides by the foot-and-mouth disease virus 2A peptide. J Biol Chem.

[17] Wang S, Yao Q, Tao J, Qiao Y, Zhang Z (2007). Co-ordinate expression of glycine betaine synthesis genes linked by the FMDV 2A region in a single open reading frame in *Pichia pastoris*. Appl Microbiol Biotechnol.

[18] Tang W, Ehrlich I, Wolff SBE, Michalski AM, Wolfl S, Hasan MT, Luthi A, Sprengel R (2009). Faithful expression of multiple proteins via 2A-peptide self-processing: a versatile and reliable method for manipulating brain circuits. J Neurosci.

[19] Lee DS, Lee KH, Jung S, Jo EJ, Han KH, Bae HJ (2012). Synergistic effects of 2A-mediated polyproteins on the production of lignocellulose degradation enzymes in tobacco plants. J Exp Bot.

[20] El Amrani A (2004). Coordinate expression and independent subcellular targeting of multiple proteins from a single transgene. Plant Physiol.

[21] Subramanian V, Schuster LA, Moore KT, Taylor LE, Baker JO, Vander Wall TA, Linger JG, Himmel ME, Decker SR (2017). A versatile 2A peptide-based bicistronic protein expressing platform for the industrial cellulase producing fungus, *Trichoderma reesei*. Biotechnol Biofuels..

[22] Guo B, Sato N, Biely P, Amano Y, Nozaki K (2016). Comparison of catalytic properties of multiple beta-glucosidases of Trichoderma reesei. Appl Microbiol Biotechnol.

[23] Bryant JA, Sellars LE, Busby SJ, Lee DJ (2014). Chromosome position effects on gene expression in Escherichia coli K-12. Nucleic Acids Res.

[24] Guangtao Z, Hartl L, Schuster A, Polak S, Schmoll M, Wang T, Seidl V, Seiboth B (2009). Gene targeting in a nonhomologous end joining deficient *Hypocrea jecorina*. J Biotechnol.

[25] Collas P, Lund EG, Oldenburg AR (2014). Closing the (nuclear) envelope on the genome: how nuclear lamins interact with promoters and modulate gene expression. BioEssays.

[26] Sousa C, de Lorenzo V, Cebolla A (1997). Modulation of gene expression through chromosomal positioning in *Escherichia coli*. Microbiology+.

[27] Gierman HJ, Indemans MH, Koster J, Goetze S, Seppen J, Geerts D, van Driel R, Versteeg R (2007). Domain-wide regulation of gene expression in the human genome. Genome Res.

[28] Bai Flagfeldt D, Siewers V, Huang L, Nielsen J (2009). Characterization of chromosomal integration sites for heterologous gene expression in *Saccharomyces cerevisiae*. Yeast.

[29] Druzhinina IS, Kubicek CP (2017). Genetic engineering of *Trichoderma reesei* cellulases and their production. Microbial Biotechnol.

[30] Grewal SI, Moazed D (2003). Heterochromatin and epigenetic control of gene expression. Science.

[31] Ottaviani A, Gilson E, Magdinier F (2008). Telomeric position effect: from the yeast paradigm to human pathologies?. Biochimie.

[32] Gilbert DM (2001). Making sense of eukaryotic DNA replication origins. Science.

[33] Lorch Y, Maier-Davis B, Kornberg RD (2014). Role of DNA sequence in chromatin remodeling and the formation of nucleosome-free regions. Genes Dev.

[34] Li C, Lin F, Li Y, Wei W, Wang H, Qin L, Zhou Z, Li B, Wu F, Chen Z (2016). A beta-glucosidase hyper-production *Trichoderma reesei* mutant reveals a potential role of cel3D in cellulase production. Microb Cell Fact.

[35] Znameroski EA, Coradetti ST, Roche CM, Tsai JC, Iavarone AT, Cate JH, Glass NL (2012). Induction of lignocellulose-degrading enzymes in *Neurospora crassa* by cellodextrins. Proc Natl Acad Sci USA.

[36] Silva-Rocha R, Castro Ldos S, Antonieto AC, Guazzaroni ME, Persinoti GF, Silva RN (2014). Deciphering the cis-regulatory elements for XYR1 and CRE1 regulators in *Trichoderma reesei*. PLoS ONE.

[37] Gruber F, Visser J, Kubicek CP, de Graaff LH (1990). The development of a heterologous transformation system for the cellulolytic fungus *Trichoderma reesei* based on a pyrG-negative mutant strain. Curr Genet.

[38] Penttilä M, Nevalainen H, Rättö M, Salminen E, Knowles J (1987). A versatile transformation system for the cellulolytic filamentous fungus *Trichoderma reesei*. Gene.

[39] Saxena R, Davidson W, Sheoran A, Giri B (2003). Purification and characterization of an alkaline thermostable lipase from *Aspergillus carneus*. Process Biochem.

[40] Kumar S, Kikon K, Upadhyay A, Kanwar SS, Gupta R (2005). Production, purification, and characterization of lipase from thermophilic and alkaliphilic *Bacillus coagulans* BTS-3. Protein Expres Purif..

[41] Bailey MJ, Tähtiharju J (2003). Efficient cellulase production by *Trichoderma reesei* in continuous cultivation on lactose medium with a computer-controlled feeding strategy. Appl Microbiol Biotechnol.

[42] Ma L, Chung WK (2014). Quantitative analysis of copy number variants based on real-time LightCycler PCR. Curr Protoc Hum Genet..

