# Supplementary material for: Identification of two integration sites in favor of transgene expression in Trichoderma reesei
Source: Biotechnol Biofuels. 2018 May 17;11:142. doi: 10.1186/s13068-018-1139-3 (PMC5956788; doi:10.1186/s13068-018-1139-3)
Supplement: Supplementary file 1 — Additional file 1. Analysis of the effect of uridine on protein secretion in strains harboring pyr4 gene. [file 13068_2018_1139_MOESM1_ESM.pdf]

Additional file 1

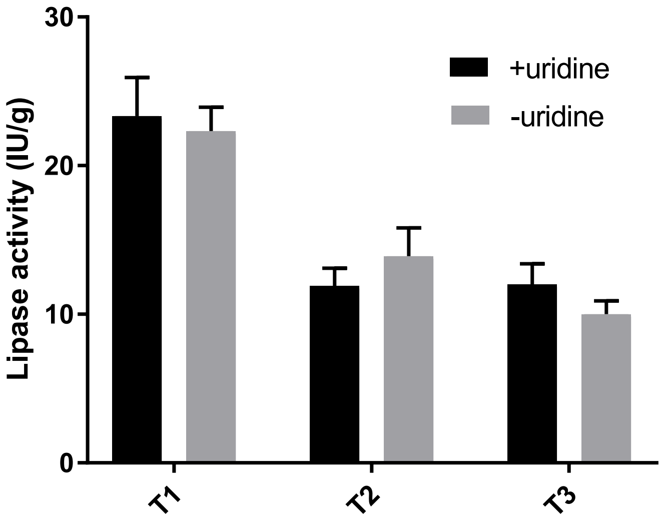


**Figure S1: Analysis of the effect of uridine on protein secretion in strains harboring *pyr4* gene.** Lipase activities of three independent transformants strains in the supernatant of 96h post-inoculation in MM media with 2% of lactose with or without 5mM uridine. All the values in the figure are the mean value of three replicates. Error bars are the standard deviation (SD) between these replicates.
