# Supplementary material for: Identification of two integration sites in favor of transgene expression in Trichoderma reesei
Source: Biotechnol Biofuels. 2018 May 17;11:142. doi: 10.1186/s13068-018-1139-3 (PMC5956788; doi:10.1186/s13068-018-1139-3)
Supplement: Supplementary file 2 — Additional file 2. The raw data of the western blot analysis of the intracellular expression of RFP and beta-actin from the mycelia lysate. [file 13068_2018_1139_MOESM2_ESM.pdf]

Additional file 2

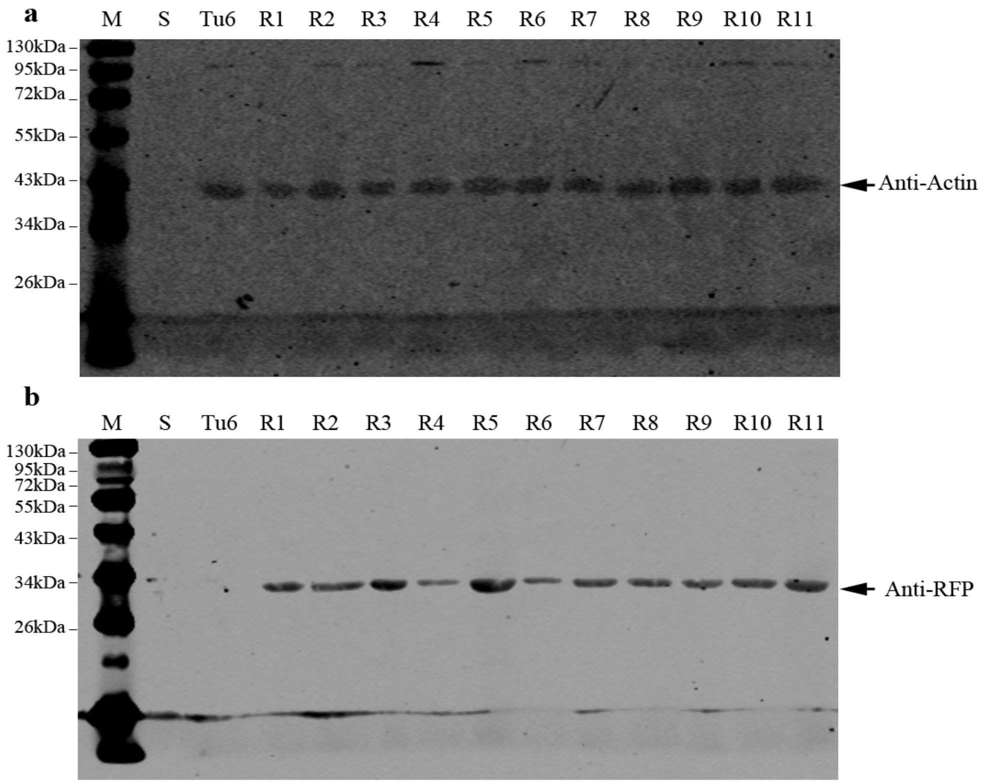

Figure S2: The raw data of the western blot analysis of the intracellular expression of beta-actin (a) and RFP (b) from the mycelia lysate. Protein samples were extracted from mycelia in 120 h cultures containing MM media with 1% Avicel as the carbon source from 24 well plates. M indicated the protein marker, S indicated the supernatant sample from the same culture.
