# Supplementary material for: Identification of two integration sites in favor of transgene expression in Trichoderma reesei
Source: Biotechnol Biofuels. 2018 May 17;11:142. doi: 10.1186/s13068-018-1139-3 (PMC5956788; doi:10.1186/s13068-018-1139-3)
Supplement: Supplementary file 3 — Additional file 3. qPCR to determine the copy number of lipA gene and cbh1 gene in the indicated strains. [file 13068_2018_1139_MOESM3_ESM.pdf]

Additional file 3

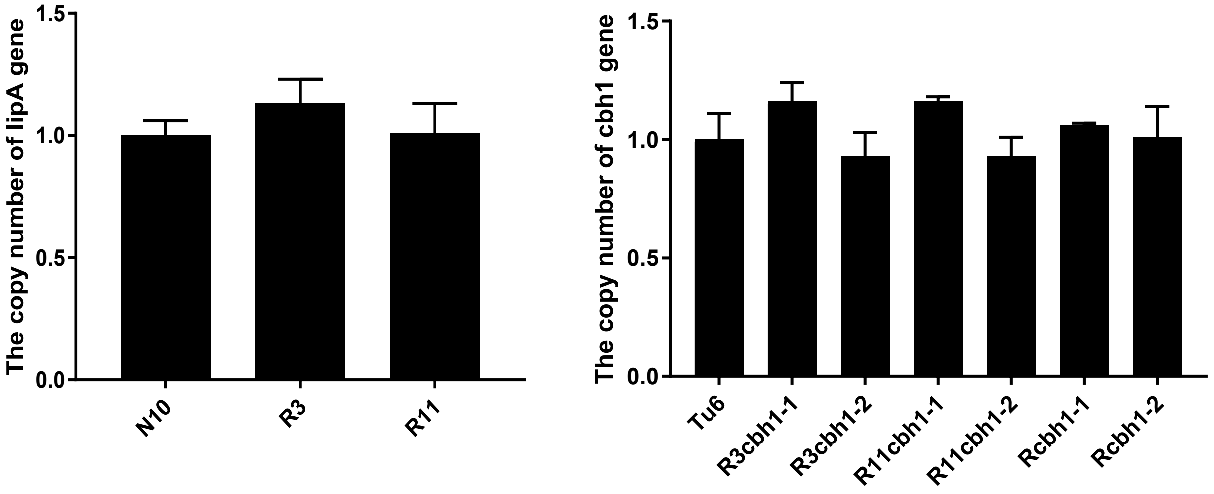

Figure S3: qPCR to determine the copy number of *lipA* gene (a) and *cbh1* gene (b) in the indicated strains. Actin gene was used as the reference gene to normalize the expression level of the test genes. N10 strain was used to quantify the copy number of *lipA* gene and Tu6 strain was used to quantify the copy number of *cbh1* gene.
