# Supplementary material for: Identification of two integration sites in favor of transgene expression in Trichoderma reesei
Source: Biotechnol Biofuels. 2018 May 17;11:142. doi: 10.1186/s13068-018-1139-3 (PMC5956788; doi:10.1186/s13068-018-1139-3)
Supplement: Supplementary file 4 — Additional file 4. Genotyping of the recovered plasmids by diagnostic PCR. [file 13068_2018_1139_MOESM4_ESM.pdf]

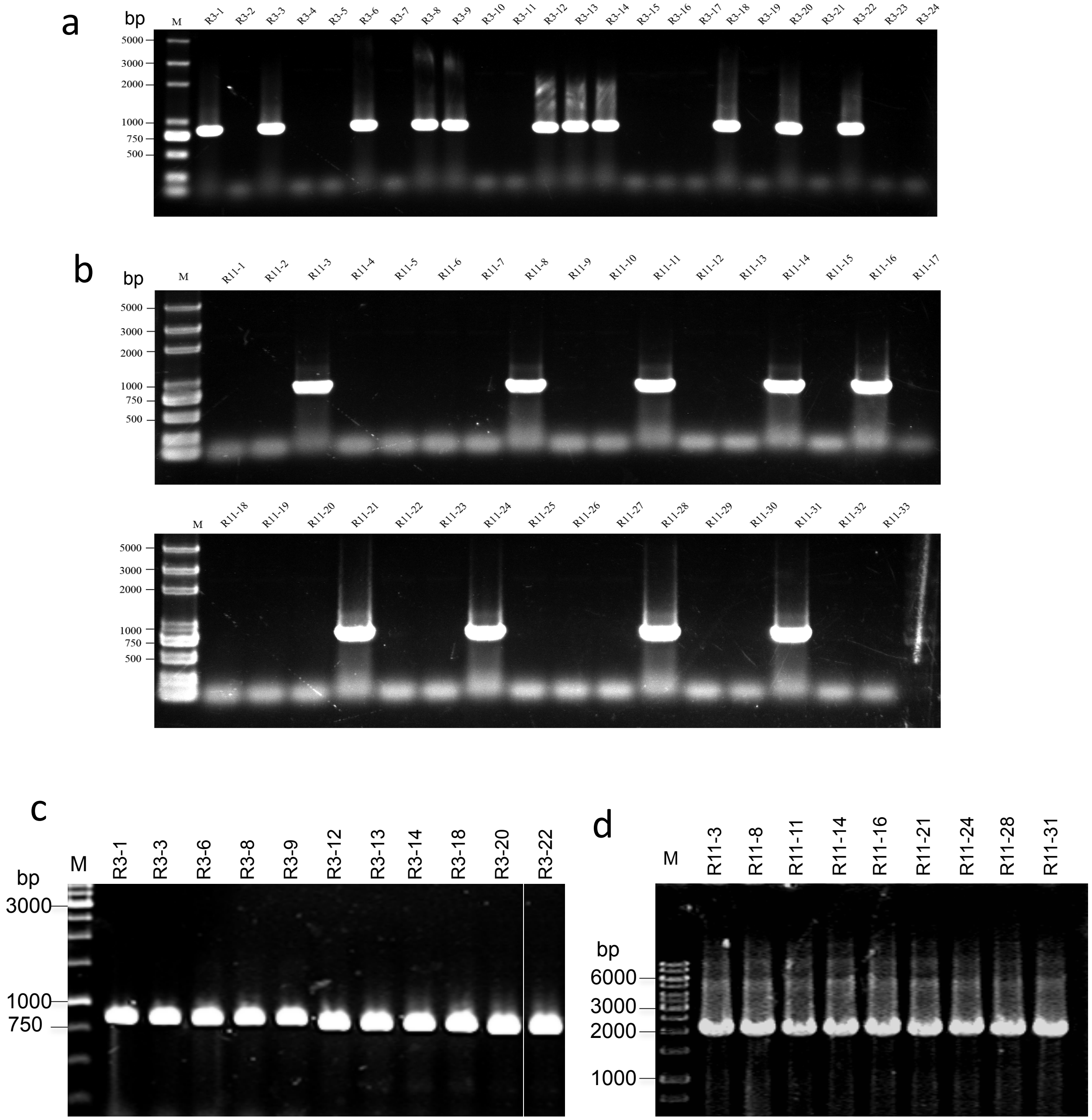

Figure S4: Genotyping of the recovered plasmids by diagnostic PCR. a, b: Colony PCR to verify the existence of lipA gene in the rescued plasmids from strain R3 (a) and R11 (b) using the primer pairs Lip-F/R, which targeted on the lipA gene. **c, d:** PCR amplification to verify the recovered plasmids form R3 (c) and R11 (d) were identical by using the primer pairs VR3-F/R and VR11-F/R respectively.
