# Supplementary material for: Identification of two integration sites in favor of transgene expression in Trichoderma reesei
Source: Biotechnol Biofuels. 2018 May 17;11:142. doi: 10.1186/s13068-018-1139-3 (PMC5956788; doi:10.1186/s13068-018-1139-3)
Supplement: Supplementary file 5 — Additional file 5. Sequence information of the integration of pSKLR in strain R3 and R11. [file 13068_2018_1139_MOESM5_ESM.docx]

**>> The whole sequence of pSKLR**

ctaaattgtaagcgttaatattttgttaaaattcgcgttaaatttttgttaaatcagctcattttttaaccaataggccgaaatcggcaaaatcccttataaatcaaaagaatagaccgagatagggttgagtgttgttccagtttggaacaagagtccactattaaagaacgtggactccaacgtcaaagggcgaaaaaccgtctatcagggcgatggcccactacgtgaaccatcaccctaatcaagttttttggggtcgaggtgccgtaaagcactaaatcggaaccctaaagggagcccccgatttagagcttgacggggaaagccggcgaacgtggcgagaaaggaagggaagaaagcgaaaggagcgggcgctagggcgctggcaagtgtagcggtcacgctgcgcgtaaccaccacacccgccgcgcttaatgcgccgctacagggcgcgtcccattcgccattcaggctgcgcaactgttgggaagggcgatcggtgcgggcctcttcgctattacgccagctggcgaaagggggatgtgctgcaaggcgattaagttgggtaacgccagggttttcccagtcacgacgttgtaaaacgacggccagtgagcgcgcgtaatacgactcactatagggcgaattgggtaccgggccccccctcgaggtcgacggacttccgcccagagctgaaggtcgcacaaccgcatgatatagggtcggcaacggcaaaaaagcacgtggctcaccgaaaagcaagatgtttgcgatctaacatccaggaacctggatacatccatcatcacgcacgaccactttgatctgctggtaaactcgtattcgccctaaaccgaagtgcgtggtaaatctacacgtgggcccctttcggtatactgcgtgtgtcttctctaggtgccattcttttcccttcctctagtgttgaattgtttgtgttggagtccgagctgtaactacctctgaatctctggagaatggtggactaacgactaccgtgcacctgcatcatgtatataatagtgatcctgagaaggggggtttggagcaatgtgggactttgatggtcatcaaacaaagaacgaagacgcctcttttgcaaagttttgtttcggctacggtgaagaactggatacttgttgtgtcttctgtgtatttttgtggcaacaagaggccagagacaatctattcaaacaccaagcttgctcttttgagctacaagaacctgtggggtatatatctagagttgtgaagtcggtaatcccgctgtatagtaatacgagtcgcatctaaatactccgaagctgctgcgaacccggagaatcgagatgtgctggaaagcttctagcgagcggctaaattagcatgaaaggctatgagaaattctggagacggcttgttgaatcatggcgttccattcttcgacaagcaaagcgttccgtcgcagtagcaggcactcattcccgaaaaaactcggagattcctaagtagcgatggaaccggaataatataataggcaatacattgagttgcctcgacggttgcaatgcaggggtactgagcttggacataactgttccgtaccccacctcttctcaacctttggcgtttccctgattcagcgtacccgtacaagtcgtaatcactattaacccagactgaccggacgtgttttgcccttcatttggagaaataatgtcattgcgatgtgtaatttgcctgcttgaccgactggggctgttcgaagcccgaatgtaggattgttatccgaactctgctcgtagaggcatgttgtgaatctgtgtcgggcaggacacgcctcgaaggttcacggcaagggaaaccaccgatagcagtgtctagtagcaacctgtaaagccgcaatgcagcatcactggaaaatacaaaccaatggctaaaagtacataagttaatgcctaaagaagtcatataccagcggctaataattgtacaatcaagtggctaaacgtaccgtaatttgccaacggcttgtggggttgcagaagcaacggcaaagccccacttccccacgtttgtttcttcactcagtccaatctcagctggtgatcccccaattgggtcgcttgtttgttccggtgaagtgaaagaagacagaggtaagaatgtctgactcggagcgttttgcatacaaccaagggcagtgatggaagacagtgaaatgttgacattcaaggagtatttagccagggatgcttgagtgtatcgtgtaaggaggtttgtctgccgatacgacgaatactgtatagtcacttctgatgaagtggtccatattgaaatgtaagtcggcactgaacaggcaaaagattgagttgaaactgcctaagatctcgggccctcgggccttcggcctttgggtgtacatgtttgtgctccgggcaaatgcaaagtgtggtaggatcgaacacactgctgcctttaccaagcagctgagggtatgtgataggcaaatgttcaggggccactgcatggtttcgaatagaaagagaagcttagccaagaacaatagccgataaagatagcctcattaaacggaatgagctagtaggcaaagtcagcgaatgtgtatatataaaggttcgaggtccgtgcctccctcatgctctccccatctactcatcaactcagatcctccaggagacttgtacaccatcttttgaggcacagaaacccaatagtcaaccgcggactgcgcatcatgtatcggaagttggccgtcatctcggccttcttggccacagctcgtgctgaattccgagtgtctcgacttccacgttggatgagctgcaattgttcgcgcaatggtctgccgcagcttattgctcgaacaatatcgactcggacgactccaacgtgacatgcacggccgacgcctgtccatcagtcgaggaagcgagcaccaagatgctgctggagtttgatttgacaaataactttggaggcacagccggtttcctggccgcggacaacaccaacaagcggctcgtggtcgccttccgaggcagtagcaccatcaagaactggattgctgatctcggcttcatcctgcaagataacgatgacctctgtactggctgcaaggttcacactggattctggaaggcatgggaagccgctgcagacaatctgacgagcaagatcaagtccgcgatgagcacgtactcaggctataccctctacttcaccgggcacagcttgggcggcgcattggctacgctgggagcaacggtcttgcgaaatgacggttatagcgttgaactgtacacctatggatgtcctcgagtcggaaactatgcgctggccgagcacatcaccagccagggatcaggagcgaacttccgcgttacacacttgaacgacatcgtcccccggttgccacccatggactttggattcagccagccaagtccagaatactggatcaccagtggcaccggagccagtgtcacggcgtcggatattgaactcatcgagggaatcaattcgacggcggggaatgcaggcgaagcaacggtggacgttttggctcacttgtggtactttttcgcgatttccgagtgcctgctaaacttcgacctcctcaagctcgccggcgacgtcgagtccaaccccggccccatggacaacaccgaggacgtcatcaaggagttcatgcagttcaaggtgcgcatggagggctccgtgaacggccactacttcgagatcgagggcgagggcgagggcaagccctacgagggcacccagaccgccaagctgcaggtgaccaagggcggccccctgcccttcgcctgggacatcctgtccccccagttccagtacggctccaaggcctacgtgaagcaccccgccgacatccccgactacatgaagctgtccttccccgagggcttcacctgggagcgctccatgaacttcgaggacggcggcgtggtggaggtgcagcaggactcctccctgcaggacggcaccttcatctacaaggtgaagttcaagggcgtgaacttccccgccgacggccccgtaatgcagaagaagactgccggctgggagccctccaccgagaagctgtacccccaggacggcgtgctgaagggcgagatctcccacgccctgaagctgaaggacggcggccactacacctgcgacttcaagaccgtgtacaaggccaagaagcccgtgcagctgcccggcaaccactacgtggactccaagctggacatcaccaaccacaacgaggactacaccgtggtggagcagtacgagcacgccgaggcccgccactccggctcccagtagactagtccagctccgtggcgaaagcctgacgcaccggtagattcttggtgagcccgtatcatgacggcggcgggagctacatggccccgggtgatttattttttttgtatctacttctgacccttttcaaatatacggtcaactcatctttcactggagatgcggcctgcttggtattgcgatgttgtcagcttggcaaattgtggctttcgaaaacacaaaacgattccttagtagccatgcattttaagataacggaatagaagaaagaggaaattaaaaaaaaaaaaaaaacaaacatcccgttcataacccgtagaatcgccgctcttcgtgtatcccagtaccacggcaaaggtatttcatgatcgttcaatgttgatattgttcccgccagtatggctccacccccatctccgcgaatctcctcttctcgaacgcggtagtggcgcgccaattggtaatgacccatagggagacaaacagcataatagcaacagtggaaattagtggcgcaataattgagaacacagtgagaccatagctggcggcctggaaagcactgttggagaccaacttgtccgttgcgaggccaacttgcattgctgtcaagacgatgacaacgtagccgaggaccgtcacaagggacgcaaagttgtcgcggatgaggtctccgtagatggcatagccggcaatccgagagtagcctctcaacaggtggccttttcgaaaccggtaaaccttgttcagacgtcctagccgcagctcaccgtaccagtatcgaggattgacggcagaatagcagtggctctccaggatttgactggacaaaatcttccagtattcccaggtcacagtgtctggcagaagtcccttctcgcgtgcgagtcgaaagtcgctatagtgcgcaatgagagcacagtaggagaataggaacccgcgagcacattgttcaatctccacatgaattggatgactgctgggcagaatgtgctgcctccaaaatcctgcgtccaacagatactctggcaggggcttcagatgaatgcctctgggcccccagataagatgcagctctggattctcggttacgatgatatcgcgagagagcacgagttggtgatggaggggacgaggaggcataggtcggccgcaggcccataaccagtcttgcacagcattgatcttcctcacgaggagctcctgatgcagaaactcctccatgttgctgattgggttgagaatttcatcgctcctggatcgtatggttgctggcaagaccctgcttaaccgtgccgtgtcatggtcatctctggtggcttcgtcgctggcctgtctttgcaattcgacagcaaatggtggagatctctctatcgtgacagtcatggtagcgatagctaggtgtcgttgcacgcacataggccgaaatgcgaagtggaaagaatttcccggcgcggaatgaagtctcgtcattttgtactcgtactcgacacctccaccgaagtgttaagaatggatccacgatgccaaaaagcttgttcatttcggctagcccgtgatcctggcgcttctagggctgaaactgtgttgttaatgtattattggctgtgtaactgacttgaatggggaatgaggagcgcgatggattcgcttgcatgtcccctggccaagacgagccgctttggcggtttgtgattcgaaggtgtgtcagcggaggcgccagggcaacacgcactgagccagccaacatgcattgctgccgacatgaatagacacgcgccgagcagacataggagacgtgttgactgtaaaaattctactgaatattagcacgcatggtctcaataagagcaataggaatgcttgccaatcataagtacgtatgtgctttttcctgcaaatggtacgtacggacagttcatgttgtctgtcatcccccactcaggctctcatgatcattttatgggactggggttttgctgactgaatggattcagccgcacgaaacaaattgggggccatgcagaagggaagcccccccagccccctgttcataatttgttaagagtcggagagctgcctagtatgaagcagcaattgataacgttgactttgcgcatgagctctgaagccgggcatatgtatcacgtttctgcctagagccgcacgggacccaagaagctcttgtcataaggtatttatgagtgttcagctgccaacgctggttctactttggctcaaccgcatcccagcggccgccaccgcggtggagctccagcttttgttccctttagtgagggttaattgcgcgcttggcgtaatcatggtcatagctgtttcctgtgtgaaattgttatccgctcacaattccacacaacatacgagccggaagcataaagtgtaaagcctggggtgcctaatgagtgagctaactcacattaattgcgttgcgctcactgcccgctttccagtcgggaaacctgtcgtgccagctgcattaatgaatcggccaacgcgcggggagaggcggtttgcgtattgggcgctcttccgcttcctcgctcactgactcgctgcgctcggtcgttcggctgcggcgagcggtatcagctcactcaaaggcggtaatacggttatccacagaatcaggggataacgcaggaaagaacatgtgagcaaaaggccagcaaaaggccaggaaccgtaaaaaggccgcgttgctggcgtttttccataggctccgcccccctgacgagcatcacaaaaatcgacgctcaagtcagaggtggcgaaacccgacaggactataaagataccaggcgtttccccctggaagctccctcgtgcgctctcctgttccgaccctgccgcttaccggatacctgtccgcctttctcccttcgggaagcgtggcgctttctcatagctcacgctgtaggtatctcagttcggtgtaggtcgttcgctccaagctgggctgtgtgcacgaaccccccgttcagcccgaccgctgcgccttatccggtaactatcgtcttgagtccaacccggtaagacacgacttatcgccactggcagcagccactggtaacaggattagcagagcgaggtatgtaggcggtgctacagagttcttgaagtggtggcctaactacggctacactagaaggacagtatttggtatctgcgctctgctgaagccagttaccttcggaaaaagagttggtagctcttgatccggcaaacaaaccaccgctggtagcggtggtttttttgtttgcaagcagcagattacgcgcagaaaaaaaggatctcaagaagatcctttgatcttttctacggggtctgacgctcagtggaacgaaaactcacgttaagggattttggtcatgagattatcaaaaaggatcttcacctagatccttttaaattaaaaatgaagttttaaatcaatctaaagtatatatgagtaaacttggtctgacagttaccaatgcttaatcagtgaggcacctatctcagcgatctgtctatttcgttcatccatagttgcctgactccccgtcgtgtagataactacgatacgggagggcttaccatctggccccagtgctgcaatgataccgcgagacccacgctcaccggctccagatttatcagcaataaaccagccagccggaagggccgagcgcagaagtggtcctgcaactttatccgcctccatccagtctattaattgttgccgggaagctagagtaagtagttcgccagttaatagtttgcgcaacgttgttgccattgctacaggcatcgtggtgtcacgctcgtcgtttggtatggcttcattcagctccggttcccaacgatcaaggcgagttacatgatcccccatgttgtgcaaaaaagcggttagctccttcggtcctccgatcgttgtcagaagtaagttggccgcagtgttatcactcatggttatggcagcactgcataattctcttactgtcatgccatccgtaagatgcttttctgtgactggtgagtactcaaccaagtcattctgagaatagtgtatgcggcgaccgagttgctcttgcccggcgtcaatacgggataataccgcgccacatagcagaactttaaaagtgctcatcattggaaaacgttcttcggggcgaaaactctcaaggatcttaccgctgttgagatccagttcgatgtaacccactcgtgcacccaactgatcttcagcatcttttactttcaccagcgtttctgggtgagcaaaaacaggaaggcaaaatgccgcaaaaaagggaataagggcgacacggaaatgttgaatactcatactcttcctttttcaatattattgaagcatttatcagggttattgtctcatgagcggatacatatttgaatgtatttagaaaaataaacaaataggggttccgcgcacatttccccgaaaagtgccac

**>> 337 bp of the flanking genomic sequence of the pSKLR construct recovered from the strain R3**

tgctgggatcgcgacaatgcctgccgagaggagagagtaagtccgcgagcgagagcacagaagaggagctgctacatacatctgcctatttgctatacagtatcttccgaagttctcacgactacccttggcaaagatggagagcccacgcatccgtccagtctgaccgacggctatataagctgcttgtcccgttcctgtgcctgatgtctatctgccgttggcctcctcatcctcatctccctgttgtctgtctcctcttagtgcttcagtgacgctaggttcggtcactttgtccccccttcgttgctctggtgtgtccaaggtctaccctgcagtggttttgaacccttgatatcctgcttgagcatccgcgtcgccatatagagcagcatattcttctatctccaaagatcccctcaccgagagttctattcacccgacccttgccttgtcatccagtccttcc

**>> The inserted sequence from pSKLR in strain R3 flanked by the PCR confirmed flanking sequenc. Sequence from pSKLR was shown in UPPERCASE.**

gggacggcggcaacggccatggcatatccacgtatcagcttcaccagctccaccaagtccgacactaccgctctcaacgttccccatcgcggcaagatcctaccggctcccgggtggaaacatgcagaggcgtcccgaaagccgggtctcggctcctgcaggatcgtcttcctccttcatccgactctgagggtggagtctggtcgacgatatccgtccgtgctcgacgttcgctacggcgtttatccgcctcaggacggaatcgaactgctttggtctgaaccacagacattgagaatcgcccacatgatctcgagggccttcgagggggcatcggggagtgtcgctcatacctcaccgaagccaagaaggctgaacttgtccatgctgagcttgtgtggagttggaccccgatcttctccaattcttttggcgggttgattgactgatagcaaacaccgattggacaggattctgccttaatcgcggcagaactctggctcatcggcttaatgccggttaaagcacggctgaaccctgttagctcaaacctgatggcggggatgatcgcggggaaactcgtccaatctggagacggtttgttcctcgtgattgattcctaccagagagggcgcagctttgctgggatcgcgacaatgcctgccgagaggagagagtaagtccgcgagcgagagcacagaagaggagctgctacatacatctgcctatttgctatacagtatcttccgaagttctcacgactacccttggcaaagatggagagcccacgcatccgtccagtctgaccgacggctatataagctgcttgtcccgttcctgtgcctgatgtctatctgccgttggcctcctcatcctcatctccctgttgtctgtctcctcttagtgcttcagtgacgctaggttcggtcactttgtccccccttcgttgctctggtgtgtccaaggtctaccctgcagtggttttgaacccttgatatcctgcttgagcatccgcgtcgccatatagagcagcatattcttctatctccaaagatcccctcaccgagagttctattcacccgacccttgccttgtcatccagtccttccGTGGCACTTTTCGGGGAAATGTGCGCGGAACCCCTATTTGTTTATTTTTCTAAATACATTCAAATATGTATCCGCTCATGAGACAATAACCCTGATAAATGCTTCAATAATATTGAAAAAGGAAGAGTATGAGTATTCAACATTTCCGTGTCGCCCTTATTCCCTTTTTTGCGGCATTTTGCCTTCCTGTTTTTGCTCACCCAGAAACGCTGGTGAAAGTAAAAGATGCTGAAGATCAGTTGGGTGCACGAGTGGGTTACATCGAACTGGATCTCAACAGCGGTAAGATCCTTGAGAGTTTTCGCCCCGAAGAACGTTTTCCAATGATGAGCACTTTTAAAGTTCTGCTATGTGGCGCGGTATTATCCCGTATTGACGCCGGGCAAGAGCAACTCGGTCGCCGCATACACTATTCTCAGAATGACTTGGTTGAGTACTCACCAGTCACAGAAAAGCATCTTACGGATGGCATGACAGTAAGAGAATTATGCAGTGCTGCCATAACCATGAGTGATAACACTGCGGCCAACTTACTTCTGACAACGATCGGAGGACCGAAGGAGCTAACCGCTTTTTTGCACAACATGGGGGATCATGTAACTCGCCTTGATCGTTGGGAACCGGAGCTGAATGAAGCCATACCAAACGACGAGCGTGACACCACGATGCCTGTAGCAATGGCAACAACGTTGCGCAAACTATTAACTGGCGAACTACTTACTCTAGCTTCCCGGCAACAATTAATAGACTGGATGGAGGCGGATAAAGTTGCAGGACCACTTCTGCGCTCGGCCCTTCCGGCTGGCTGGTTTATTGCTGATAAATCTGGAGCCGGTGAGCGTGGGTCTCGCGGTATCATTGCAGCACTGGGGCCAGATGGTAAGCCCTCCCGTATCGTAGTTATCTACACGACGGGGAGTCAGGCAACTATGGATGAACGAAATAGACAGATCGCTGAGATAGGTGCCTCACTGATTAAGCATTGGTAACTGTCAGACCAAGTTTACTCATATATACTTTAGATTGATTTAAAACTTCATTTTTAATTTAAAAGGATCTAGGTGAAGATCCTTTTTGATAATCTCATGACCAAAATCCCTTAACGTGAGTTTTCGTTCCACTGAGCGTCAGACCCCGTAGAAAAGATCAAAGGATCTTCTTGAGATCCTTTTTTTCTGCGCGTAATCTGCTGCTTGCAAACAAAAAAACCACCGCTACCAGCGGTGGTTTGTTTGCCGGATCAAGAGCTACCAACTCTTTTTCCGAAGGTAACTGGCTTCAGCAGAGCGCAGATACCAAATACTGTCCTTCTAGTGTAGCCGTAGTTAGGCCACCACTTCAAGAACTCTGTAGCACCGCCTACATACCTCGCTCTGCTAATCCTGTTACCAGTGGCTGCTGCCAGTGGCGATAAGTCGTGTCTTACCGGGTTGGACTCAAGACGATAGTTACCGGATAAGGCGCAGCGGTCGGGCTGAACGGGGGGTTCGTGCACACAGCCCAGCTTGGAGCGAACGACCTACACCGAACTGAGATACCTACAGCGTGAGCTATGAGAAAGCGCCACGCTTCCCGAAGGGAGAAAGGCGGACAGGTATCCGGTAAGCGGCAGGGTCGGAACAGGAGAGCGCACGAGGGAGCTTCCAGGGGGAAACGCCTGGTATCTTTATAGTCCTGTCGGGTTTCGCCACCTCTGACTTGAGCGTCGATTTTTGTGATGCTCGTCAGGGGGGCGGAGCCTATGGAAAAACGCCAGCAACGCGGCCTTTTTACGGTTCCTGGCCTTTTGCTGGCCTTTTGCTCACATGTTCTTTCCTGCGTTATCCCCTGATTCTGTGGATAACCGTATTACCGCCTTTGAGTGAGCTGATACCGCTCGCCGCAGCCGAACGACCGAGCGCAGCGAGTCAGTGAGCGAGGAAGCGGAAGAGCGCCCAATACGCAAACCGCCTCTCCCCGCGCGTTGGCCGATTCATTAATGCAGCTGGCACGACAGGTTTCCCGACTGGAAAGCGGGCAGTGAGCGCAACGCAATTAATGTGAGTTAGCTCACTCATTAGGCACCCCAGGCTTTACACTTTATGCTTCCGGCTCGTATGTTGTGTGGAATTGTGAGCGGATAACAATTTCACACAGGAAACAGCTATGACCATGATTACGCCAAGCAAGGTATTTATGAGTGTTCAGCTGCCAACGCTGGTTCTACTTTGGCTCAACCGCATCCCATATGACAAGAGCTTCTTGGGTCCCGTGCGGCTCTAGGCAGAAACGTGATACATATGCCCGGCTTCAGAGCTCATGCGCAAAGTCAACGTTATCAATTGCTGCTTCATACTAGGCAGCTCTCCGACTCTTAACAAATTATGAACAGGGGGCTGGGGGGGCTTCCCTTCTGCATGGCCCCCAATTTGTTTCGTGCGGCTGAATCCATTCAGTCAGCAAAACCCCAGTCCCATAAAATGATCATGAGAGCCTGAGTGGGGGATGACAGACAACATGAACTGTCCGTACGTACCATTTGCAGGAAAAAGCACATACGTACTTATGATTGGCAAGCATTCCTATTGCTCTTATTGAGACCATGCGTGCTAATATTCAGTAGAATTTTTACAGTCAACACGTCTCCTATGTCTGCTCGGCGCGTGTCTATTCATGTCGGCAGCAATGCATGTTGGCTGGCTCAGTGCGTGTTGCCCTGGCGCCTCCGCTGACACACCTTCGAATCACAAACCGCCAAAGCGGCTCGTCTTGGCCAGGGGACATGCAAGCGAATCCATCGCGCTCCTCATTCCCCATTCAAGTCAGTTACACAGCCAATAATACATTAACAACACAGTTTCAGCCCTAGAAGCGCCAGGATCACGGGCTAGCCGAAATGAACAAGCTTTTTGGCATCGTGGATCCATTCTTAACACTTCGGTGGAGGTGTCGAGTACGAGTACAAAATGACGAGACTTCATTCCGCGCCGGGAAATTCTTTCCACTTCGCATTTCGGCCTATGTGCGTGCAACGACACCTAGCTATCGCTACCATGACTGTCACGATAGAGAGATCTCCACCATTTGCTGTCGAATTGCAAAGACAGGCCAGCGACGAAGCCACCAGAGATGACCATGACACGGCACGGTTAAGCAGGGTCTTGCCAGCAACCATACGATCCAGGAGCGATGAAATTCTCAACCCAATCAGCAACATGGAGGAGTTTCTGCATCAGGAGCTCCTCGTGAGGAAGATCAATGCTGTGCAAGACTGGTTATGGGCCTGCGGCCGACCTATGCCTCCTCGTCCCCTCCATCACCAACTCGTGCTCTCTCGCGATATCATCGTAACCGAGAATCCAGAGCTGCATCTTATCTGGGGGCCCAGAGGCATTCATCTGAAGCCCCTGCCAGAGTATCTGTTGGACGCAGGATTTTGGAGGCAGCACATTCTGCCCAGCAGTCATCCAATTCATGTGGAGATTGAACAATGTGCTCGCGGGTTCCTATTCTCCTACTGTGCTCTCATTGCGCACTATAGCGACTTTCGACTCGCACGCGAGAAGGGACTTCTGCCAGACACTGTGACCTGGGAATACTGGAAGATTTTGTCCAGTCAAATCCTGGAGAGCCACTGCTATTCTGCCGTCAATCCTCGATACTGGTACGGTGAGCTGCGGCTAGGACGTCTGAACAAGGTTTACCGGTTTCGAAAAGGCCACCTGTTGAGAGGCTACTCTCGGATTGCCGGCTATGCCATCTACGGAGACCTCATCCGCGACAACTTTGCGTCCCTTGTGACGGTCCTCGGCTACGTTGTCATCGTCTTGACAGCAATGCAAGTTGGCCTCGCAACGGACAAGTTGGTCTCCAACAGTGCTTTCCAGGCCGCCAGCTATGGTCTCACTGTGTTCTCAATTATTGCGCCACTAATTTCCACTGTTGCTATTATGCTGTTTGTCTCCCTATGGGTCATTACCAATTGGCGCGCCACTACCGCGTTCGAGAAGAGGAGATTCGCGGAGATGGGGGTGGAGCCATACTGGCGGGAACAATATCAACATTGAACGATCATGAAATACCTTTGCCGTGGTACTGGGATACACGAAGAGCGGCGATTCTACGGGTTATGAACGGGATGTTTGTTTTTTTTTTTTTTTTAATTTCCTCTTTCTTCTATTCCGTTATCTTAAAATGCATGGCTACTAAGGAATCGTTTTGTGTTTTCGAAAGCCACAATTTGCCAAGCTGACAACATCGCAATACCAAGCAGGCCGCATCTCCAGTGAAAGATGAGTTGACCGTATATTTGAAAAGGGTCAGAAGTAGATACAAAAAAAATAAATCACCCGGGGCCATGTAGCTCCCGCCGCCGTCATGATACGGGCTCACCAAGAATCTACCGGTGCGTCAGGCTTTCGCCACGGAGCTGGACTAGTCTACTGGGAGCCGGAGTGGCGGGCCTCGGCGTGCTCGTACTGCTCCACCACGGTGTAGTCCTCGTTGTGGTTGGTGATGTCCAGCTTGGAGTCCACGTAGTGGTTGCCGGGCAGCTGCACGGGCTTCTTGGCCTTGTACACGGTCTTGAAGTCGCAGGTGTAGTGGCCGCCGTCCTTCAGCTTCAGGGCGTGGGAGATCTCGCCCTTCAGCACGCCGTCCTGGGGGTACAGCTTCTCGGTGGAGGGCTCCCAGCCGGCAGTCTTCTTCTGCATTACGGGGCCGTCGGCGGGGAAGTTCACGCCCTTGAACTTCACCTTGTAGATGAAGGTGCCGTCCTGCAGGGAGGAGTCCTGCTGCACCTCCACCACGCCGCCGTCCTCGAAGTTCATGGAGCGCTCCCAGGTGAAGCCCTCGGGGAAGGACAGCTTCATGTAGTCGGGGATGTCGGCGGGGTGCTTCACGTAGGCCTTGGAGCCGTACTGGAACTGGGGGGACAGGATGTCCCAGGCGAAGGGCAGGGGGCCGCCCTTGGTCACCTGCAGCTTGGCGGTCTGGGTGCCCTCGTAGGGCTTGCCCTCGCCCTCGCCCTCGATCTCGAAGTAGTGGCCGTTCACGGAGCCCTCCATGCGCACCTTGAACTGCATGAACTCCTTGATGACGTCCTCGGTGTTGTCCATGGGGCCGGGGTTGGACTCGACGTCGCCGGCGAGCTTGAGGAGGTCGAAGTTTAGCAGGCACTCGGAAATCGCGAAAAAGTACCACAAGTGAGCCAAAACGTCCACCGTTGCTTCGCCTGCATTCCCCGCCGTCGAATTGATTCCCTCGATGAGTTCAATATCCGACGCCGTGACACTGGCTCCGGTGCCACTGGTGATCCAGTATTCTGGACTTGGCTGGCTGAATCCAAAGTCCATGGGTGGCAACCGGGGGACGATGTCGTTCAAGTGTGTAACGCGGAAGTTCGCTCCTGATCCCTGGCTGGTGATGTGCTCGGCCAGCGCATAGTTTCCGACTCGAGGACATCCATAGGTGTACAGTTCAACGCTATAACCGTCATTTCGCAAGACCGTTGCTCCCAGCGTAGCCAATGCGCCGCCCAAGCTGTGCCCGGTGAAGTAGAGGGTATAGCCTGAGTACGTGCTCATCGCGGACTTGATCTTGCTCGTCAGATTGTCTGCAGCGGCTTCCCATGCCTTCCAGAATCCAGTGTGAACCTTGCAGCCAGTACAGAGGTCATCGTTATCTTGCAGGATGAAGCCGAGATCAGCAATCCAGTTCTTGATGGTGCTACTGCCTCGGAAGGCGACCACGAGCCGCTTGTTGGTGTTGTCCGCGGCCAGGAAACCGGCTGTGCCTCCAAAGTTATTTGTCAAATCAAACTCCAGCAGCATCTTGGTGCTCGCTTCCTCGACTGATGGACAGGCGTCGGCCGTGCATGTCACGTTGGAGTCGTCCGAGTCGATATTGTTCGAGCAATAAGCTGCGGCAGACCATTGCGCGAACAATTGCAGCTCATCCAACGTGGAAGTCGAGACACTCGGAATTCAGCACGAGCTGTGGCCAAGAAGGCCGAGATGACGGCCAACTTCCGATACATGATGCGCAGTCCGCGGTTGACTATTGGGTTTCTGTGCCTCAAAAGATGGTGTACAAGTCTCCTGGAGGATCTGAGTTGATGAGTAGATGGGGAGAGCATGAGGGAGGCACGGACCTCGAACCTTTATATATACACATTCGCTGACTTTGCCTACTAGCTCATTCCGTTTAATGAGGCTATCTTTATCGGCTATTGTTCTTGGCTAAGCTTCTCTTTCTATTCGAAACCATGCAGTGGCCCCTGAACATTTGCCTATCACATACCCTCAGCTGCTTGGTAAAGGCAGCAGTGTGTTCGATCCTACCACACTTTGCATTTGCCCGGAGCACAAACATGTACACCCAAAGGCCGAAGGCCCGAGGGCCCGAGATCTTAGGCAGTTTCAACTCAATCTTTTGCCTGTTCAGTGCCGACTTACATTTCAATATGGACCACTTCATCAGAAGTGACTATACAGTATTCGTCGTATCGGCAGACAAACCTCCTTACACGATACACTCAAGCATCCCTGGCTAAATACTCCTTGAATGTCAACATTTCACTGTCTTCCATCACTGCCCTTGGTTGTATGCAAAACGCTCCGAGTCAGACATTCTTACCTCTGTCTTCTTTCACTTCACCGGAACAAACAAGCGACCCAATTGGGGGATCACCAGCTGAGATTGGACTGAGTGAAGAAACAAACGTGGGGAAGTGGGGCTTTGCCGTTGCTTCTGCAACCCCACAAGCCGTTGGCAAATTACGGTACGTTTAGCCACTTGATTGTACAATTATTAGCCGCTGGTATATGACTTCTTTAGGCATTAACTTATGTACTTTTAGCCATTGGTTTGTATTTTCCAGTGATGCTGCATTGCGGCTTTACAGGTTGCTACTAGACACTGCTATCGGTGGTTTCCCTTGCCGTGAACCTTCGAGGCGTGTCCTGCCCGACACAGATTCACAACATGCCTCTACGAGCAGAGTTCGGATAACAATCCTACATTCGGGCTTCGAACAGCCCCAGTCGGTCAAGCAGGCAAATTACACATCGCAATGACATTATTTCTCCAAATGAAGGGCAAAACACGTCCGGTCAGTCTGGGTTAATAGTGATTACGACTTGTACGGGTACGCTGAATCAGGGAAACGCCAAAGGTTGAGAAGAGGTGGGGTACGGAACAGTTATGTCCAAGCTCAGTACCCCTGCATTGCAACCGTCGAGGCAACTCAATGTATTGCCTATTATATTATTCCGGTTCCATCGCTACTTAGGAATCTCCGAGTTTTTTCGGGAATGAGTGCCTGCTACTGCGACGGAACGCTTTGCTTGTCGAAGAATGGAACGCCATGATTCAACAAGCCGTCTCCAGAATTTCTCATAGCCTTTCATGCTAATTTAGCCGCTCGCTAGAAGCTTTCCAGCACATCTCGATTCTCCGGGTTCGCAGCAGCTTCGGAGTATTTAGATGCGACTCGTATTACTATACAGCGGGATTACCGACTTCACAACTCTAGATATATACCCCACAGGTTCTTGTAGCTCAAAAGAGCAAGCTTGGTGTTTGAATAGATTGTCTCTGGCCTCTTGTTGCCACAAAAATACACAGAAGACACAACAAGTATCCAGTTCTTCACCGTAGCCGAAACAAAACTTTGCAAAAGAGGCGTCTTCGTTCTTTGTTTGATGACCATCAAAGTCCCACATTGCTCCAAACCCCCCTTCTCAGGATCACTATTATATACATGATGCAGGTGCACGGTAGTCGTTAGTCCACCATTCTCCAGAGATTCAGAGGTAGTTACAGCTCGGACTCCAACACAAACAATTCAACACTAGAGGAAGGGAAAAGAATGGCACCTAGAGAAGACACACGCAGTATACCGAAAGGGGCCCACGTGTAGATTTACCACGCACTTCGGTTGGTACCCAATTCGCCCTATAGTGAGTCGTATTACGCGCGCTCACTGGCCGTCGTTTTACAACGTCGTGACTGGGAAAACCCTGGCGTTACCCAACTTAATCGCCTTGCAGCACATCCCCCTTTCGCCAGCTGGCGTAATAGCGAAGAGGCCCGCACCGATCGCCCTTCCCAACAGTTGCGCAGCCTGAATGGCGAATGGGACGCGCCCTGTAGCGGCGCATTAAGCGCGGCGGGTGTGGTGGTTACGCGCAGCGTGACCGCTACACTTGCCAGCGCCCTAGCGCCCGCTCCTTTCGCTTTCTTCCCTTCCTTTCTCGCCACGTTCGCCGGCTTTCCCCGTCAAGCTCTAAATCGGGGGCTCCCTTTAGGGTTCCGATTTAGTGCTTTACGGCACCTCGACCCCAAAAAACTTGATTAGGGTGATGGTTCACGTAGTGGGCCATCGCCCTGATAGACGGTTTTTCGCCCTTTGACGTTGGAGTCCACGTTCTTTAATAGTGGACTCTTGTTCCAAACTGGAACAACACTCAACCCTATCTCGGTCTATTCTTTTGATTTATAAGGGATTTTGCCGATTTCGGCCTATTGGTTAAAAAATGAGCTGATTTAACAAAAATTTAACGCGAATTTTAACAAAATATTAACGCTTACAATTTatggctgatattgatgttgaggccatcttgaagaagctcaccctggccgagaaggtcgatctgctggctggtgagttgtcacgctatttgatagttgttcatttacgccatgtcccaaaatctacatagcaatgaccgaacattcaatctggctccctatatcgaatcattgtctgactgaacaaggtatcgacttctggcacacaaaggctctccccaagcatggagtcccctctctccgctttacagatggccccaacggcgtaagagggaccaagttcttcaatggcgtccctgcggcctgcttcccttgcggcacgtcgctcggttccacattcaaccaaactctgctcgaagaggcaggtaagatgatgggcaaagaggccatcgctaagagtgcgcatgtgatcctcggcccgactatcaacatgcaacgctcccctctcggtggacgtggcttcgagtcgattggtgaggatccgttcctggcgggcttgggagctgcggctctcatccgcggcattcagagcactggagtgcaggctacgatcaagcactttttgtgcaatgatcaggaggacaggcgcatgatggtgcagagcatcgtcacggagcgggctctccgtgaaatctacgcactcccgttccagattgctgtgcgagactcccagccgggtgcgttcatgacggcgtacaatggcatcaatggcgtgtcgtgcagcgagaaccctaaatatcttgatgggatgcttcgaaaggaatggggttgggatggcctaatcatgagcgactggtacggcacatacagtaccacagaagccgttgtggcaggcctcgacctcgagatgcccggacctccacgcttccgaggagaaacactcaagttcaacgtctccaacggaaagccctttatccacgtcattgaccagagggctagggaagttcttcagttcgtcaagaagtgtgctgcctccggagtgacggagaacggccccgagacgactgtcaacaacacccccgaaacggcagctctcctccggaaggttggcaacgagggcatcgtgctgctgaagaacgagaacaacgttctgcccttgagcaagaagaagaagacgctgattgtcggccccaacgccaagcaggccacataccacggcggaggctctgccgcactcagggcctactacgcagtcactccctttgacggcctcagcaagcagctcgagacgccgccatcgtacaccgtcggcgcctacacccaccgcttcctccccatt

**248 bp of the flanking genomic sequence of the pSKLR construct recovered from the strain R11**

ctataaattgggatgaaaatcacgcagaagtcaatgctcgtttctcttccttttcacatctcgttcatcgttcttccaccaagaaagaatcaaaagtcaactcaatcgtaacctatccaaataaaagtattcagtgatatacttcacaccccccctccaaccccctctctccaatgcatctgacacacagcaggccaaataccacactctcctgtcggtacaacctcgtccgtcgccatggcgtcctt

**>> The inserted sequence from pSKLR in strain R11 flanked by the PCR confirmed flanking sequence. Sequence from pSKLR was shown in UPPERCASE.**

caggttgatgctgtcatgaatgctgtgcccgccgaggcagaggtcattttggcgtatgagcctgtatgggcgattggtgccagtcaacctgctggagagaagcacatcctcgatgttgttgctgggatcagagggttggagtccgttgggaggagggcggggacgacgagggttttgtatggggggagtgccgggccggggctctacgagaagttgaagagcggactggacggattgttcctggggcggttcggacacgatccggagcagtttgtcaagacgatacaggaagtagctgaagcgtgagggagagcaatgaactatctcttgggatggctgtgatgacaggctgctccgtatgataggtacagtatgtacgaagaagtactactattacaattcttcatcaaggctgattgttgctaggtacacaaaggccattactcagagcgaagcccaattgcccatcccccatagatcctctcttgagaagaatgaaatggatcagcattgacctcaactcccatgatccagtcaacggctccaaacaagtggcacaatggcccaatccgtccaagtttttagggtcctcttcaactatccccgagtccaccacaactttcttagcaaataatatcttcacgataacacacacacacagccctaaattctctcatggctaaaatcacacgcccggataccaccggatcatttcccgcccggcttttggccgttgaatgtttgcttccaactgccgacatcgccgtcactatgattgtatcgagagccgatcaattggcccttacccctccttttttaaatgtgacggttggtgagaatccggtcgactataaattgggatgaaaatcacgcagaagtcaatgctcgtttctcttccttttcacatctcgttcatcgttcttccaccaagaaagaatcaaaagtcaactcaatcgtaacctatccaaataaaagtattcagtgatatacttcacaccccccctccaaccccctctctccaatgcatctgacacacagcaggccaaataccacactctcctgtcggtacaacctcgtccgtcgccatggcgtccttCTCGAGGGGGGGCCCGGTACCCAATTCGCCCTATAGTGAGTCGTATTACGCGCGCTCACTGGCCGTCGTTTTACAACGTCGTGACTGGGAAAACCCTGGCGTTACCCAACTTAATCGCCTTGCAGCACATCCCCCTTTCGCCAGCTGGCGTAATAGCGAAGAGGCCCGCACCGATCGCCCTTCCCAACAGTTGCGCAGCCTGAATGGCGAATGGGACGCGCCCTGTAGCGGCGCATTAAGCGCGGCGGGTGTGGTGGTTACGCGCAGCGTGACCGCTACACTTGCCAGCGCCCTAGCGCCCGCTCCTTTCGCTTTCTTCCCTTCCTTTCTCGCCACGTTCGCCGGCTTTCCCCGTCAAGCTCTAAATCGGGGGCTCCCTTTAGGGTTCCGATTTAGTGCTTTACGGCACCTCGACCCCAAAAAACTTGATTAGGGTGATGGTTCACGTAGTGGGCCATCGCCCTGATAGACGGTTTTTCGCCCTTTGACGTTGGAGTCCACGTTCTTTAATAGTGGACTCTTGTTCCAAACTGGAACAACACTCAACCCTATCTCGGTCTATTCTTTTGATTTATAAGGGATTTTGCCGATTTCGGCCTATTGGTTAAAAAATGAGCTGATTTAACAAAAATTTAACGCGAATTTTAACAAAATATTAACGCTTACAATTTAGGTGGCACTTTTCGGGGAAATGTGCGCGGAACCCCTATTTGTTTATTTTTCTAAATACATTCAAATATGTATCCGCTCATGAGACAATAACCCTGATAAATGCTTCAATAATATTGAAAAAGGAAGAGTATGAGTATTCAACATTTCCGTGTCGCCCTTATTCCCTTTTTTGCGGCATTTTGCCTTCCTGTTTTTGCTCACCCAGAAACGCTGGTGAAAGTAAAAGATGCTGAAGATCAGTTGGGTGCACGAGTGGGTTACATCGAACTGGATCTCAACAGCGGTAAGATCCTTGAGAGTTTTCGCCCCGAAGAACGTTTTCCAATGATGAGCACTTTTAAAGTTCTGCTATGTGGCGCGGTATTATCCCGTATTGACGCCGGGCAAGAGCAACTCGGTCGCCGCATACACTATTCTCAGAATGACTTGGTTGAGTACTCACCAGTCACAGAAAAGCATCTTACGGATGGCATGACAGTAAGAGAATTATGCAGTGCTGCCATAACCATGAGTGATAACACTGCGGCCAACTTACTTCTGACAACGATCGGAGGACCGAAGGAGCTAACCGCTTTTTTGCACAACATGGGGGATCATGTAACTCGCCTTGATCGTTGGGAACCGGAGCTGAATGAAGCCATACCAAACGACGAGCGTGACACCACGATGCCTGTAGCAATGGCAACAACGTTGCGCAAACTATTAACTGGCGAACTACTTACTCTAGCTTCCCGGCAACAATTAATAGACTGGATGGAGGCGGATAAAGTTGCAGGACCACTTCTGCGCTCGGCCCTTCCGGCTGGCTGGTTTATTGCTGATAAATCTGGAGCCGGTGAGCGTGGGTCTCGCGGTATCATTGCAGCACTGGGGCCAGATGGTAAGCCCTCCCGTATCGTAGTTATCTACACGACGGGGAGTCAGGCAACTATGGATGAACGAAATAGACAGATCGCTGAGATAGGTGCCTCACTGATTAAGCATTGGTAACTGTCAGACCAAGTTTACTCATATATACTTTAGATTGATTTAAAACTTCATTTTTAATTTAAAAGGATCTAGGTGAAGATCCTTTTTGATAATCTCATGACCAAAATCCCTTAACGTGAGTTTTCGTTCCACTGAGCGTCAGACCCCGTAGAAAAGATCAAAGGATCTTCTTGAGATCCTTTTTTTCTGCGCGTAATCTGCTGCTTGCAAACAAAAAAACCACCGCTACCAGCGGTGGTTTGTTTGCCGGATCAAGAGCTACCAACTCTTTTTCCGAAGGTAACTGGCTTCAGCAGAGCGCAGATACCAAATACTGTCCTTCTAGTGTAGCCGTAGTTAGGCCACCACTTCAAGAACTCTGTAGCACCGCCTACATACCTCGCTCTGCTAATCCTGTTACCAGTGGCTGCTGCCAGTGGCGATAAGTCGTGTCTTACCGGGTTGGACTCAAGACGATAGTTACCGGATAAGGCGCAGCGGTCGGGCTGAACGGGGGGTTCGTGCACACAGCCCAGCTTGGAGCGAACGACCTACACCGAACTGAGATACCTACAGCGTGAGCTATGAGAAAGCGCCACGCTTCCCGAAGGGAGAAAGGCGGACAGGTATCCGGTAAGCGGCAGGGTCGGAACAGGAGAGCGCACGAGGGAGCTTCCAGGGGGAAACGCCTGGTATCTTTATAGTCCTGTCGGGTTTCGCCACCTCTGACTTGAGCGTCGATTTTTGTGATGCTCGTCAGGGGGGCGGAGCCTATGGAAAAACGCCAGCAACGCGGCCTTTTTACGGTTCCTGGCCTTTTGCTGGCCTTTTGCTCACATGTTCTTTCCTGCGTTATCCCCTGATTCTGTGGATAACCGTATTACCGCCTTTGAGTGAGCTGATACCGCTCGCCGCAGCCGAACGACCGAGCGCAGCGAGTCAGTGAGCGAGGAAGCGGAAGAGCGCCCAATACGCAAACCGCCTCTCCCCGCGCGTTGGCCGATTCATTAATGCAGCTGGCACGACAGGTTTCCCGACTGGAAAGCGGGCAGTGAGCGCAACGCAATTAATGTGAGTTAGCTCACTCATTAGGCACCCCAGGCTTTACACTTTATGCTTCCGGCTCGTATGTTGTGTGGAATTGTGAGCGGATAACAATTTCACACAGGAAACAGCTATGACCATGATTACGCCAAGCGCGCAATTAACCCTCACTAAAGGGAACAAAAGCTGGAGCTCCACCGCGGTGGCGGCCGCTGGGATGCGGTTGAGCCAAAGTAGAACCAGCGTTGGCAGCTGAACACTCATAAATACCTTATGACAAGAGCTTCTTGGGTCCCGTGCGGCTCTAGGCAGAAACGTGATACATATGCCCGGCTTCAGAGCTCATGCGCAAAGTCAACGTTATCGCTGGCTCAGTGCGTGTTGCCCTGGCGCCTCCGCTGACACACCTTCGAATCACAAACCGCCAAAGCGGCTCGTCTTGGCCAGGGGACATGCAAGCGAATCCATCGCGCTCCTCATTCCCCATTCAAGTCAGTTACACAGCCAATAATACATTAACAACACAGTTTCAGCCCTAGAAGCGCCAGGATCACGGGCTAGCCGAAATGAACAAGCTTTTTGGCATCGTGGATCCATTCTTAACACTTCGGTGGAGGTGTCGAGTACGAGTACAAAATGACGAGACTTCATTCCGCGCCGGGAAATTCTTTCCACTTCGCATTTCGGCCTATGTGCGTGCAACGACACCTAGCTATCGCTACCATGACTGTCACGATAGAGAGATCTCCACCATTTGCTGTCGAATTGCAAAGACAGGCCAGCGACGAAGCCACCAGAGATGACCATGACACGGCACGGTTAAGCAGGGTCTTGCCAGCAACCATACGATCCAGGAGCGATGAAATTCTCAACCCAATCAGCAACATGGAGGAGTTTCTGCATCAGGAGCTCCTCGTGAGGAAGATCAATGCTGTGCAAGACTGGTTATGGGCCTGCGGCCGACCTATGCCTCCTCGTCCCCTCCATCACCAACTCGTGCTCTCTCGCGATATCATCGTAACCGAGAATCCAGAGCTGCATCTTATCTGGGGGCCCAGAGGCATTCATCTGAAGCCCCTGCCAGAGTATCTGTTGGACGCAGGATTTTGGAGGCAGCACATTCTGCCCAGCAGTCATCCAATTCATGTGGAGATTGAACAATGTGCTCGCGGGTTCCTATTCTCCTACTGTGCTCTCATTGCGCACTATAGCGACTTTCGACTCGCACGCGAGAAGGGACTTCTGCCAGACACTGTGACCTGGGAATACTGGAAGATTTTGTCCAGTCAAATCCTGGAGAGCCACTGCTATTCTGCCGTCAATCCTCGATACTGGTACGGTGAGCTGCGGCTAGGACGTCTGAACAAGGTTTACCGGTTTCGAAAAGGCCACCTGTTGAGAGGCTACTCTCGGATTGCCGGCTATGCCATCTACGGAGACCTCATCCGCGACAACTTTGCGTCCCTTGTGACGGTCCTCGGCTACGTTGTCATCGTCTTGACAGCAATGCAAGTTGGCCTCGCAACGGACAAGTTGGTCTCCAACAGTGCTTTCCAGGCCGCCAGCTATGGTCTCACTGTGTTCTCAATTATTGCGCCACTAATTTCCACTGTTGCTATTATGCTGTTTGTCTCCCTATGGGTCATTACCAATTGGCGCGCCACTACCGCGTTCGAGAAGAGGAGATTCGCGGAGATGGGGGTGGAGCCATACTGGCGGGAACAATATCAACATTGAACGATCATGAAATACCTTTGCCGTGGTACTGGGATACACGAAGAGCGGCGATTCTACGGGTTATGAACGGGATGTTTGTTTTTTTTTTTTTTTTAATTTCCTCTTTCTTCTATTCCGTTATCTTAAAATGCATGGCTACTAAGGAATCGTTTTGTGTTTTCGAAAGCCACAATTTGCCAAGCTGACAACATCGCAATACCAAGCAGGCCGCATCTCCAGTGAAAGATGAGTTGACCGTATATTTGAAAAGGGTCAGAAGTAGATACAAAAAAAATAAATCACCCGGGGCCATGTAGCTCCCGCCGCCGTCATGATACGGGCTCACCAAGAATCTACCGGTGCGTCAGGCTTTCGCCACGGAGCTGGACTAGTCTACTGGGAGCCGGAGTGGCGGGCCTCGGCGTGCTCGTACTGCTCCACCACGGTGTAGTCCTCGTTGTGGTTGGTGATGTCCAGCTTGGAGTCCACGTAGTGGTTGCCGGGCAGCTGCACGGGCTTCTTGGCCTTGTACACGGTCTTGAAGTCGCAGGTGTAGTGGCCGCCGTCCTTCAGCTTCAGGGCGTGGGAGATCTCGCCCTTCAGCACGCCGTCCTGGGGGTACAGCTTCTCGGTGGAGGGCTCCCAGCCGGCAGTCTTCTTCTGCATTACGGGGCCGTCGGCGGGGAAGTTCACGCCCTTGAACTTCACCTTGTAGATGAAGGTGCCGTCCTGCAGGGAGGAGTCCTGCTGCACCTCCACCACGCCGCCGTCCTCGAAGTTCATGGAGCGCTCCCAGGTGAAGCCCTCGGGGAAGGACAGCTTCATGTAGTCGGGGATGTCGGCGGGGTGCTTCACGTAGGCCTTGGAGCCGTACTGGAACTGGGGGGACAGGATGTCCCAGGCGAAGGGCAGGGGGCCGCCCTTGGTCACCTGCAGCTTGGCGGTCTGGGTGCCCTCGTAGGGCTTGCCCTCGCCCTCGCCCTCGATCTCGAAGTAGTGGCCGTTCACGGAGCCCTCCATGCGCACCTTGAACTGCATGAACTCCTTGATGACGTCCTCGGTGTTGTCCATGGGGCCGGGGTTGGACTCGACGTCGCCGGCGAGCTTGAGGAGGTCGAAGTTTAGCAGGCACTCGGAAATCGCGAAAAAGTACCACAAGTGAGCCAAAACGTCCACCGTTGCTTCGCCTGCATTCCCCGCCGTCGAATTGATTCCCTCGATGAGTTCAATATCCGACGCCGTGACACTGGCTCCGGTGCCACTGGTGATCCAGTATTCTGGACTTGGCTGGCTGAATCCAAAGTCCATGGGTGGCAACCGGGGGACGATGTCGTTCAAGTGTGTAACGCGGAAGTTCGCTCCTGATCCCTGGCTGGTGATGTGCTCGGCCAGCGCATAGTTTCCGACTCGAGGACATCCATAGGTGTACAGTTCAACGCTATAACCGTCATTTCGCAAGACCGTTGCTCCCAGCGTAGCCAATGCGCCGCCCAAGCTGTGCCCGGTGAAGTAGAGGGTATAGCCTGAGTACGTGCTCATCGCGGACTTGATCTTGCTCGTCAGATTGTCTGCAGCGGCTTCCCATGCCTTCCAGAATCCAGTGTGAACCTTGCAGCCAGTACAGAGGTCATCGTTATCTTGCAGGATGAAGCCGAGATCAGCAATCCAGTTCTTGATGGTGCTACTGCCTCGGAAGGCGACCACGAGCCGCTTGTTGGTGTTGTCCGCGGCCAGGAAACCGGCTGTGCCTCCAAAGTTATTTGTCAAATCAAACTCCAGCAGCATCTTGGTGCTCGCTTCCTCGACTGATGGACAGGCGTCGGCCGTGCATGTCACGTTGGAGTCGTCCGAGTCGATATTGTTCGAGCAATAAGCTGCGGCAGACCATTGCGCGAACAATTGCAGCTCATCCAACGTGGAAGTCGAGACACTCGGAATTCAGCACGAGCTGTGGCCAAGAAGGCCGAGATGACGGCCAACTTCCGATACATGATGCGCAGTCCGCGGTTGACTATTGGGTTTCTGTGCCTCAAAAGATGGTGTACAAGTCTCCTGGAGGATCTGAGTTGATGAGTAGATGGGGAGAGCATGAGGGAGGCACGGACCTCGAACCTTTATATATACACATTCGCTGACTTTGCCTACTAGCTCATTCCGTTTAATGAGGCTATCTTTATCGGCTATTGTTCTTGGCTAAGCTTCTCTTTCTATTCGAAACCATGCAGTGGCCCCTGAACATTTGCCTATCACATACCCTCAGCTGCTTGGTAAAGGCAGCAGTGTGTTCGATCCTACCACACTTTGCATTTGCCCGGAGCACAAACATGTACACCCAAAGGCCGAAGGCCCGAGGGCCCGAGATCTTAGGCAGTTTCAACTCAATCTTTTGCCTGTTCAGTGCCGACTTACATTTCAATATGGACCACTTCATCAGAAGTGACTATACAGTATTCGTCGTATCGGCAGACAAACCTCCTTACACGATACACTCAAGCATCCCTGGCTAAATACTCCTTGAATGTCAACATTTCACTGTCTTCCATCACTGCCCTTGGTTGTATGCAAAACGCTCCGAGTCAGACATTCTTACCTCTGTCTTCTTTCACTTCACCGGAACAAACAAGCGACCCAATTGGGGGATCACCAGCTGAGATTGGACTGAGTGAAGAAACAAACGTGGGGAAGTGGGGCTTTGCCGTTGCTTCTGCAACCCCACAAGCCGTTGGCAAATTACGGTACGTTTAGCCACTTGATTGTACAATTATTAGCCGCTGGTATATGACTTCTTTAGGCATTAACTTATGTACTTTTAGCCATTGGTTTGTATTTTCCAGTGATGCTGCATTGCGGCTTTACAGGTTGCTACTAGACACTGCTATCGGTGGTTTCCCTTGCCGTGAACCTTCGAGGCGTGTCCTGCCCGACACAGATTCACAACATGCCTCTACGAGCAGAGTTCGGATAACAATCCTACATTCGGGCTTCGAACAGCCCCAGTCGGTCAAGCAGGCAAATTACACATCGCAATGACATTATTTCTCCAAATGAAGGGCAAAACACGTCCGGTCAGTCTGGGTTAATAGTGATTACGACTTGTACGGGTACGCTGAATCAGGGAAACGCCAAAGGTTGAGAAGAGGTGGGGTACGGAACAGTTATGTCCAAGCTCAGTACCCCTGCATTGCAACCGTCGAGGCAACTCAATGTATTGCCTATTATATTATTCCGGTTCCATCGCTACTTAGGAATCTCCGAGTTTTTTCGGGAATGAGTGCCTGCTACTGCGACGGAACGCTTTGCTTGTCGAAGAATGGAACGCCATGATTCAACAAGCCGTCTCCAGAATTTCTCATAGCCTTTCATGCTAATTTAGCCGCTCGCTAGAAGCTTTCCAGCACATCTCGATTCTCCGGGTTCGCAGCAGCTTCGGAGTATTTAGATGCGACTCGTATTACTATACAGCGGGATTACCGACTTCACAACTCTAGATATATACCCCACAGGTTCTTGTAGCTCAAAAGAGCAAGCTTGGTGTTTGAATAGATTGTCTCTGGCCTCTTGTTGCCACAAAAATACACAGAAGACACAACAAGTATCCAGTTCTTCACCGTAGCCGAAACAAAACTTTGCAAAAGAGGCGTCTTCGTTCTTTGTTTGATGACCATCAAAGTCCCACATTGCTCCAAACCCCCCTTCTCAGGATCACTATTATATACATGATGCAGGTGCACGGTAGTCGTTAGTCCACCATTCTCCAGAGATTCAGAGGTAGTTACAGCTCGGACTCCAACACAAACAATTCAACACTAGAGGAAGGGAAAAGAATGGCACCTAGAGAAGACACACGCAGTATACCGAAAGGGGCCCACGTGTAGATTTACCACGCACTTCGGTTTAGGGCGAATACGAGTTTACCAGCAGATCAAAGTGGTCGTGCGTGATGATGGATGTATCCAGGTTCCTGGATGTTAGATCGCAAACATCTTGCTTTTCGGTGAGCCACGTGCTTTTTTGCCGTTGCCGACCCTATATCATGCGGTTGTGCGACCTTCAGCTCTGGGCGGAAGTCCGTCGACggtagtccgtccagacggggttctgtctcctcatcttcaggtaatgtgtatcgcatgagccggtgcccgggctcccccccttcctttgtcccacttcactcctctcccctcgtctctgcttgtctttacaaacaaggaaacacacccaatgagatggagaaggtgattgtgtggaaataaggaagagggggctcgggctgagaatatacggccctgaacttgatctggataataccagcgaaaggatcatgctcctcccccttttcaatcccgtccaaatccctaacccttgtaggcccatcatgagccctcccgccgccgtctcgcccccccagcgaaccgctgaactcgtcaccccgtccaagatggccgttgcccagccgcagcagcacctcgaggcccaggccaagtccgtctcggacatgttcggccagtgggactcgttcaccttctcgcccatccgcgagtcccaggtgtcgcgcgccatgacccgccgctacttcgaggacctcgaccgctacgccgagtccgacattgtcatcatcggcgccggctcctgcggcctcagcaccgcctacgtcctcggcacccagcgcccggacctcaagattgccatcatcgaggcctccgtctcccccggcggcggtgcctggctgggcggccagctcttctccgccatggtcatgcgcaagcctgccgatgccttcctccgcgagattggcgtcccgtacgaggacgagggcaactacgtcgtcgtcaagcacgccgccctcttcacctccaccatcatggccaaggtgctgcagctacccaacgtcaagctcttcaacgccacctgcgtcgaagacctcatcacccgcccctctgccgagggcgtgcgcatcgccggtgtcgtcaccaactggaccctcgtctccatgcaccacgacgaccagtcctgcatggaccccaacaccatcaacgcgcccctcgtcatctccaccaccggccacgacggccccatgggtgccttc
