# Supplementary material for: Identification of two integration sites in favor of transgene expression in Trichoderma reesei
Source: Biotechnol Biofuels. 2018 May 17;11:142. doi: 10.1186/s13068-018-1139-3 (PMC5956788; doi:10.1186/s13068-018-1139-3)
Supplement: Supplementary file 6 — Additional file 6. Analysis of the conservation of the identified R3 and R11 loci in other Trichoderma species. [file 13068_2018_1139_MOESM6_ESM.docx]

**Table S1: The list of sequenced Trichoderma sp. strains**

| Name | Assembly Length | # Genes | Published |
| --- | --- | --- | --- |
| *Trichoderma reesei* RUT C-30 v1.0 | 32,689,233 | 9,852 | Jourdier E et al., 2017 |
| *Trichoderma reesei* v2.0 | 33,454,791 | 9,143 | Martinez D et al., 2008 |
| *Trichoderma asperellum* CBS 433.97 v1.0 | 37,464,610 | 12,586 |  |
| *Trichoderma asperellum* TR356 v1.0 | 35,388,325 | 12,320 |  |
| *Trichoderma atroviride* v2.0 | 36,143,664 | 11,828 | Kubicek CP et al., 2011 |
| *Trichoderma citrinoviride* TUCIM 6016 v4.0 | 33,215,161 | 9,737 |  |
| *Trichoderma gamsii* T6085 | 37,970,415 | 10,944 | Baroncelli R et al., 2016 |
| *Trichoderma harzianum* CBS 226.95 v1.0 | 40,980,678 | 14,095 |  |
| *Trichoderma harzianum* TR274 v1.0 | 40,870,399 | 13,932 |  |
| *Trichoderma longibrachiatum* ATCC 18648 v3.0 | 32,238,325 | 10,938 |  |
| *Trichoderma virens* Gv29-8 v2.0 | 39,022,666 | 12,423 | [Kubicek CP et al., 2011](https://www.ncbi.nlm.nih.gov/pubmed/21501500) |

**Table S2: Sequence alignment of the R3 and R11 insertion sites**

| Subject Organism | Query Sequence | Subject Sequence | Query Coverage (%) | Percent of Identity (%) | E-value |
| --- | --- | --- | --- | --- | --- |
| *Trichoderma reesei* QM6a | R3 | chromosome IV | 100 | 100 | 0.0 |
|  | R11 | chromosome II | 100 | 100 | 0.0 |
| *Trichoderma reesei* RUT C-30 v1.0 | R3 | scaffold_34 | 100 | 100 | 0.0 |
|  | R11 | scaffold_27 | 100 | 100 | 0.0 |
| *Trichoderma reesei* v2.0 | R3 | scaffold_33 | 100 | 100 | 0.0 |
|  | R11 | scaffold_25 | 100 | 100 | 0.0 |
| *Trichoderma asperellum* CBS 433.97 v1.0 | R3 | scaffold_3 | 42 | 86.37 | 0.0 |
|  | R11 | scaffold_10 | 30 | 81.87 | 0.0 |
| *Trichoderma asperellum* TR356 v1.0 | R3 | scaffold_1477 | 42 | 86.49 | 0.0 |
|  | R11 | scaffold_5 | 34 | 80.51 | 0.0 |
| *Trichoderma atroviride* v2.0 | R3 | contig_25 | 42 | 86.59 | 0.0 |
|  | R11 | contig_24 | 31 | 84.97 | 0.0 |
| *Trichoderma citrinoviride* TUCIM 6016 v4.0 | R3 | scaffold_19 | 98 | 81.59 | 0.0 |
|  | R11 | scaffold_8 | 87 | 89.97 | 0.0 |
| *Trichoderma gamsii* T6085 | R3 | scaffold_125 | 42 | 86.64 | 0.0 |
|  | R11 | scaffold_26 | 34 | 81.59 | 0.0 |
| Trichoderma harzianum CBS 226.95 v1.0 | R3 | scaffold_16 | 42 | 88.61 | 0.0 |
|  | R11 | scaffold_13 | 54 | 84.69 | 0.0 |
| Trichoderma harzianum TR274 v1.0 | R3 | scaffold_324 | 42 | 88.61 | 0.0 |
|  | R11 | scaffold_6 | 54 | 84.69 | 0.0 |
| *Trichoderma longibrachiatum* ATCC 18648 v3.0 | R3 | scaffold_25 | 75 | 85.57 | 0.0 |
|  | R11 | scaffold_12 | 88 | 89.23 | 0.0 |
| *Trichoderma virens* Gv29-8 v2.0 | R3 | scaffold_5 | 42 | 87.31 | 0.0 |
|  | R11 | scaffold_1 | 40 | 81.92 | 0.0 |
