# Supplementary material for: Identification of two integration sites in favor of transgene expression in Trichoderma reesei
Source: Biotechnol Biofuels. 2018 May 17;11:142. doi: 10.1186/s13068-018-1139-3 (PMC5956788; doi:10.1186/s13068-018-1139-3)
Supplement: Supplementary file 7 — Additional file 7. Primers used in this study. [file 13068_2018_1139_MOESM7_ESM.docx]

**Table S3. Primers used in this study.**

| **Primer name** | **Primer sequence(5' to 3')** |  |
| --- | --- | --- |
| **Primers used qRT-PCR study** | |  |
| qRT-actin-F | TGAGAGCGGTGGTATCCACG | |
| qRT-actin-R | GGTACCACCAGACATGACAATGTTG | |
| qRT-lipase-F | GCAAGATCAAGTCCGCGATG | |
| qRT-lipase-R | CCAGCGCATAGTTTCCGACT | |
| qRT-cbh1-F | TACCTTATGGCGAGCGACAC | |
| qRT-cbh1-R | GCGTCAGGGGTTCATGGTAA | |
| **Primers used in the generation of Plasmid pSKLR** | |  |
| Pcbh1-F | GCGTCGACAACCGAAGTGCGTGGTAAATCTACA | |
| Pcbh1-R | CGGAATTCAGCACGAGCTGTGGCCAAGAAGG | |
| Tcbh1-F | GGACTAGTAGCTCCGTGGCGAAAGCCTGACG | |
| Tcbh1-R | TAAAGCGGCCGCTGGGATGCGGTTGAGCCAAAGTAG | |
| lip2a-F | CGGAATTCCACCACCACCACCACCACAGTGTCTCGACTTCCACGTTGGAT | |
| lip2a-R | GGGGCCGGGGTTGGACTCGACGTCGCCGGCGAGCTTGAGGAGGTCGAAGTT TAGCAGGCACTCGGAAATCGCG | |
| 2ared-F | AACTTCGACCTCCTCAAGCTCGCCGGCGACGTCGAGTCCAACCCCGGCCC  CATGGACAACACCGAGGACGTCATC | |
| 2ared-R | GGACTAGTCTACTGGGAGCCGGAGTGGCGG | |
| **Primers used in the generation of strains with integration of Pcbh1-cbh1-Tcbh2 into R3 locus** | | |
| Mlup-F | GTATCGATAAGCTTGATATCGACAAGGAAACGGTAGCGGAAAG | |
| Mlup-R | GGAACACTACGAGAAACGGCCAACGGCCAAAAGCCGGGC | |
| Pcbh1-2F | CCCTTCGTTGCTCTGGTGTGTCGACTTCCGCCCAGAGCTGAAGGT | |
| cbh1-2R | GACAGACCAGAGGCAAGTCAACGCTTTACAGGCACTGAGAGTAGTAAGG | |
| Tcbh2-F | CCTTACTACTCTCAGTGCCTGTAAAGCGTTGACTTGCCTCTGGTCTGTC | |
| R3Tcbh2-R | CTCAAGCAGGATATCAAGGGTTCCGGGAGCAGCGACGCAACGCCGAC | |
| Mldown-F | GTCGGCGTTGCGTCGCTGCTCCCGGAACCCTTGATATCCTGCTTGAG | |
| Mldown-R | GTGGATCCCCCGGGCTGCAGGAACGTGCTCCTTGGCGAGGGCGAC | |
| MVec-F | GTCGCCCTCGCCAAGGAGCACGTTCCTGCAGCCCGGGGGATCCAC | |
| MVec-R | GCTTTCCGCTACCGTTTCCTTGTCGATATCAAGCTTATCGATAC | |
| Ocbh1-F | CTTGAGGCATGATGGATGACACG | |
| OM29-F | AGTGTAAAGAGCAATAATGCG | |
| Vcbh1-R | CGTCTGCCAGCTTGGATAAGAAGCTG | |
| **Primers used in the generation of strains with integration of Pcbh1-cbh1-Tcbh2 into R3 locus** | | |
| Flup-F | GTATCGATAAGCTTGATATCGAGACACGGTAGACAAGCGAATC | |
| Flup-R | GGAACACTACGAGAAACGGCCAACGGCCAAAAGCCGGGC | |
| Pcbh1-2F | GCCCGGCTTTTGGCCGTTGGCCGTTTCTCGTAGTGTTCC | |
| R11Tcbh2-R | CTTCTGCGTGATTTTCATCCGGGAGCAGCGACGCAACGCCGAC | |
| Fldown-F | GTCGGCGTTGCGTCGCTGCTCCCGGATGAAAATCACGCAGAAG | |
| Fldown-R | GATCCCCCGGGCTGCAGGAATCGTGCTCAAGCATGAGCTTGC | |
| Vec-F | GCAAGCTCATGCTTGAGCACGATTCCTGCAGCCCGGGGGATC | |
| Vec-R | GATTCGCTTGTCTACCGTGTCTCGATATCAAGCTTATCGATAC | |
| Ond1-F | AGTGTAAAGAGCAATAATGCG | |
| **Primers used in generation of cbh1 gene deletion strain** | | |
| Dcbh1-1F | AGACCGGCAGCGGCCGCAAATCTACACGTGGGCCCCTTTCGG | |
| Dcbh1-1R | AGGATGGTTTGGATGCAGTTGGATGCGCAGTCCGCGGTTGAC | |
| Dcbh1-2F | AACCGCGGACTGCGCATCCAACTGCATCCAAACCATCCTAC | |
| Dcbh1-2R | TCAGGCTTTCGCCACGGAGCTCCCCAAAGTCGCAATATCGG | |
| Dcbh1-3F | ACCCGATATTGCGACTTTGGGGAGCTCCGTGGCGAAAGCCTG | |
| Dcbh1-3R | AGCACTGCGAGGGGCCGTTTCGTGCGGCTGAATCCATTCAG | |
| Dcbh1-4F | TTCAGCCGCACGAAACGGCCCCTCGCAGTGCTCCAATTTTC | |
| Dcbh1-4R | AAAGGGGCCCACGTGTAGATTTGCGGCCGCTGCCGGTCTCC | |
| **Primers used for sequencing the recovered plasmid from R3 and R11 genome** | | |
| R3-F1 | GCAACTTTATCCGCCTCCAT | |
| R3-R1 | TAGCTTCCCGGCAACAATTAAT | |
| R3-F2 | TTGAATGTATTTAGAAAAAT | |
| R3-R2 | GTGGCGATAAGTCGTGTCTT | |
| R3-F3 | TGAATTGTAATACGACTCACT | |
| R3-R3 | TGTGTGGAATTGTGAGCGGAT | |
| R3-F4 | TGCAATGCAGGGGTACTGAGCT | |
| R3-R4 | TTGTCAAGTGGATAGAGCTCT | |
| R3-F5 | TAGCCAGGGATGCTTGAGTGT | |
| R3-R5 | ATGGGTTCCAGTCGCAGCCA | |
| R3-F6 | TGCTACGATGGCAACACTT | |
| **Primers used for genotyping of the recovered plasmids by diagnostic PCR** | | |
| Lip-F | AGTGTCTCGACTTCCACGTTG | |
| Lip-R | TAGCACCCACTCGGAAATC | |
| VR3-F | TGCTGGGATCGCGACAATGCC | |
| VR3-R | GTGACTGGTGAGTACTCAAC | |
| VR11-F | CTATAAATTGGGATGAAAATC | |
| VR11-R | GATTTTGGTCATGAGATTATC | |
